# Harnessing a previously unidentified capability of bacterial allosteric transcription factors for sensing diverse small molecules in vitro

**DOI:** 10.1126/sciadv.aau4602

**Published:** 2018-11-28

**Authors:** Jiaqian Cao, Yongpeng Yao, Keqiang Fan, Gaoyi Tan, Wensheng Xiang, Xuekui Xia, Shanshan Li, Weishan Wang, Lixin Zhang

**Affiliations:** 1 State Key Laboratory of Microbial Resources and Chinese Academy of Sciences (CAS) Key Laboratory of Pathogenic Microbiology and Immunology, Institute of Microbiology, CAS, Beijing 100101, P.R. China.; 2 State Key Laboratory of Bioreactor Engineering, East China University of Science and Technology, Shanghai 200237, P.R. China.; 3 State Key Laboratory for Biology of Plant Diseases and Insect Pests, Institute of Plant Protection, Chinese Academy of Agricultural Sciences, Beijing 100193, P.R. China.; 4 Key Laboratory for Biosensor of Shandong Province, Biology Institute, Qilu University of Technology (Shandong Academy of Sciences), Jinan 250013, P.R. China.; 5 Laboratory for Marine Biology and Biotechnology, Qingdao National Laboratory for Marine Science and Technology, Qingdao 266061, P.R. China.

## Abstract

A plethora of bacterial allosteric transcription factors (aTFs) have been identified to sense a variety of small molecules. Introduction of a novel aTF-based approach to sense diverse small molecules in vitro will signify a broad series of detection applications. Here, we found that aTFs could interact with their nicked DNA binding sites. Building from this new finding, we designed and implemented a novel aTF-based nicked DNA template–assisted signal transduction system (aTF-NAST) by using the competition between aTFs and T4 DNA ligase to bind to the nicked DNA. This aTF-NAST could reliably and modularly transduce the signal of small molecules recognized by aTFs to the ligated DNA signal, thus enabling the small molecules to be measured via various mature and robust DNA detection methods. Coupling this aTF-NAST with three DNA detection methods, we demonstrated nine novel biosensors for the detection of an antiseptic 4-hydroxybenzoic acid, a disease marker uric acid and an antibiotic tetracycline. These biosensors show impressive sensitivity and robustness in real-life analysis, highlighting the great potential of our aTF-NAST for biosensing applications.

## Teaser

We develop a novel biosensing platform for the detection of diverse small molecules.

## INTRODUCTION

Development of small-molecule biosensing approaches with the characteristics of simplicity, efficiency, reliability, and low cost is of paramount importance for bioanalytical applications in areas as diverse as scientific research, environmental monitoring, food safety, and clinical diagnosis (*1*). Signal recognition and signal transduction are the two essential aspects for the development of any biosensors (*2*). Currently available biorecognition elements for small molecules include enzymes, antibodies, nucleic acids (e.g., aptamer), and so on; each of these must be coupled to some form of signal transduction and/or amplification system that ultimately facilitate measurement (*3*). However, these available biorecognition elements only sense a limited number of small molecules. Given the increasing detection requirements of diverse small molecules, the development of novel and universal biosensing approach for a new type of recognition elements will boost the construction of diverse biosensors (*1*, *4*).

Bacteria have evolved a plethora of allosteric transcription factors (aTFs) to sense a wide range of small molecules (*5*). These aTFs usually comprise an effector binding domain (EBD) and a DNA binding domain (DBD) (*5*): Typically, the binding of a specific small molecule to the EBD induces a conformational change that can either attenuate or enhance the affinity of DBD for the aTF binding site (TFBS) (*5*). As genetic parts, aTFs have been co-opted for use, as gene expression switches in many synthetic biological applications in vivo (*6*–*9*). For the detection of small molecules, many whole-cell biosensors (WCBs) were constructed by coupling the aTF-based genetic circuits with reporter genes (*5*, *10*–*12*). However, given the extreme molecular complexity of cells and the sensitivity of living systems to variations in experimental and environmental conditions, these WCBs have, to date, suffered from poor robustness and from issues with reproducibility when used to detect the small-molecule analytes (*10*). Inspired by the allosteric effect of aTFs that enables them to both receive and transduce signals of small molecules, we recently demonstrated that aTFs could be used as a new type of recognition element in a homogeneous buffer in vitro, which could avoid the drawbacks of aTF-based WCBs (*13*, *14*). Plenty of aTFs with high specificity to diverse effectors (*15*) give us an opportunity to develop biosensors for the in vitro detection of diverse small molecules, especially those that are unable to be sensed by conventional recognition elements (such as enzymes, antibodies, and aptamers). However, the recognition mechanism of aTFs is quite different from the widely used conventional recognition elements. Although surface plasmon resonance, surface-enhanced Raman scattering, and microfluidics-based approaches may be amendable to aTF recognition elements, the developed biosensors coupling these approaches are instrument dependent and difficult to be used for on-site detection. Therefore, it is an urgent need to develop a universal and stable signal transduction system to transduce the signal of small molecules sensed by aTFs to the signal that can be easily measured by mature detection methods. To address this issue, here, we aimed to design and implement a convenient and robust signal transduction system for the development of aTF-based small-molecule biosensors in vitro.

DNA signals are very stable and are convenient to be accurately quantified in both laboratory and on-site detection. A variety of mature and robust DNA detection methods are used ubiquitously throughout life science and clinical medicine, such as already commercialized real-time quantitative polymerase chain reaction (RT-qPCR) (*16*), digital PCR (*17*), loop-mediated isothermal amplification (LAMP) (*18*), rolling circle amplification (RCA) (*19*), recombinase polymerase amplification (RPA) (*20*), and recently reported CRISPR-Cas–mediated supersensitive nucleic acid detection methods (*21*–*24*). We therefore consider that a reliable signal transduction system can be designed to transduce the small-molecule recognition of aTFs to a DNA signal.

To conveniently and faithfully transduce small-molecule signals to a DNA signal in vitro, here, starting from our proposed concept of an aTF-based nicked DNA template–assisted signal transduction system (aTF-NAST), we first characterized a previously unidentified capability of aTFs—interacting with their nicked TFBSs in vitro. Then, using this new capability of aTFs, the aTF-NAST was established to transduce the signal of small molecules sensed by aTFs to the ligated DNA signal. Such a design enables any of the mature and standard DNA detection methods to be compatible to this aTF-NAST. As a proof of concept, we coupled this system with three widely used DNA detection methods [RT-qPCR (*16*), RCA (*19*), and RPA (*20*)] and demonstrated nine novel biosensors for the detection of an antiseptic 4-hydroxybenzoic acid (4-HBA), a disease marker uric acid (UA), and an antibiotic tetracycline (TC) with impressive sensitivity and stability. For context, 4-HBA is an antiseptic used in foods and cosmetics and is also a common environmental pollutant (*25*). To our knowledge, there are few biosensing methods for 4-HBA detection up to now. UA is a key biomarker for the diagnosis of several diseases including gout and leukemia (*26*). TC is commonly used as an animal feed additive, and health protection regulations demand frequent testing of residual TC levels in foods, livestock, and soil (*27*, *28*). By benchmarking the performance of the developed biosensors to the state-of-the-art ones constructed by other approaches, our biosensors show promising robustness and sensitivity in testing authentic samples. Overall, our work presents a new route for developing aTF-based small-molecule biosensors and highlights the great potential of aTF-NAST for biosensing applications.

## RESULTS

### Principle of the aTF-NAST

**Fig. 1:**
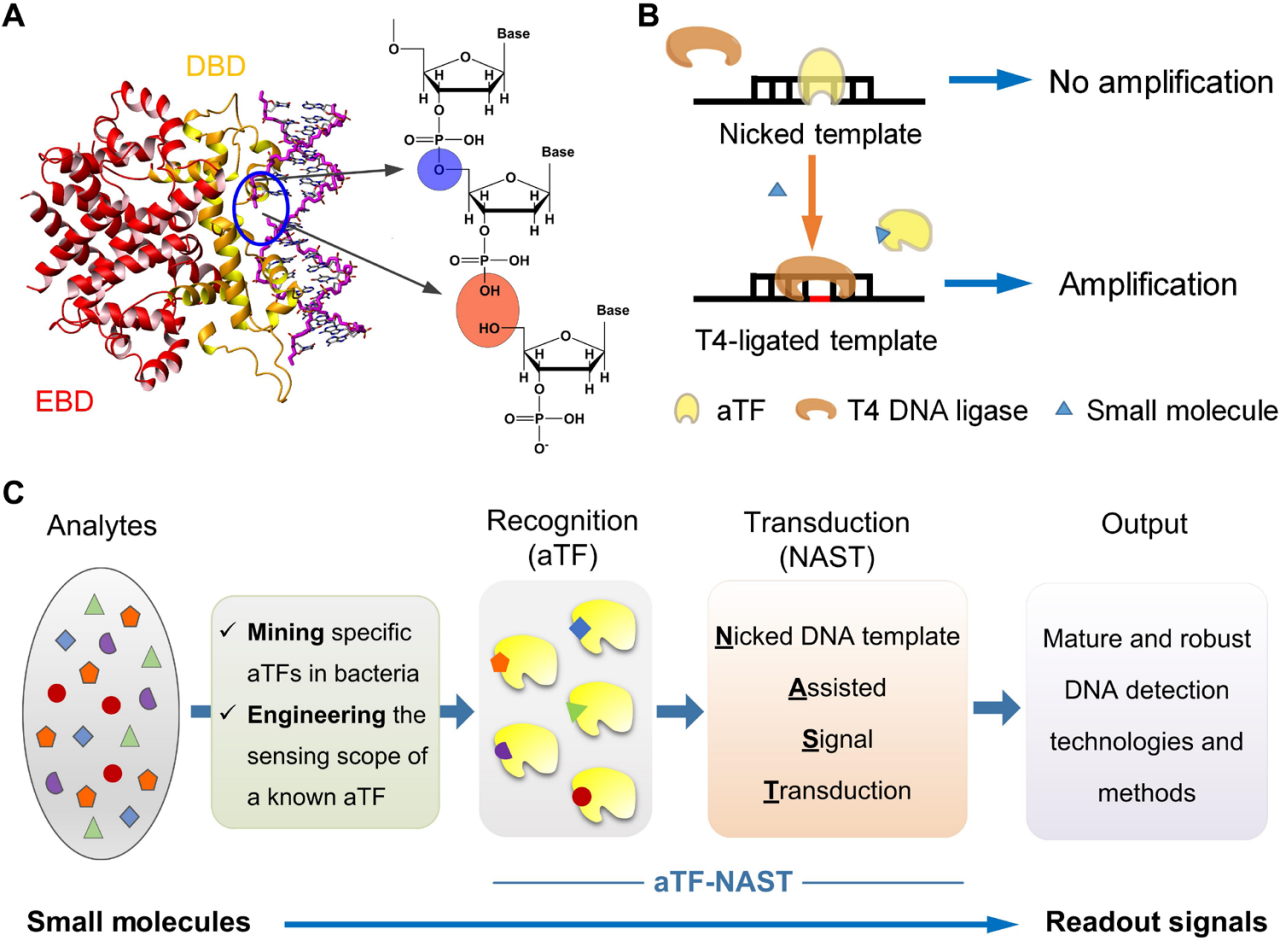
The aTF-NAST. (**A**) Schematic for the interaction between an aTF and its TFBS with a nick. Intact and broken phosphodiester bonds are highlighted in blue and orange circles, respectively. (**B**) Schematic of the aTF-NAST. (**C**) Workflow for the development of small-molecule biosensors using aTF-NAST.

The DBD of a given aTF specifically binds to the conserved palindromic TFBS (*29*). Our design started from the idea that perhaps a single nick (a phosphodiester bond break) in TFBS might not cause major disruptive effects on the binding of an aTF (Fig. 1A). If this is the case, then we further hypothesized that the aTF would be able to compete with T4 DNA ligase to bind to the nicked TFBS in vitro (Fig. 1A), just as with the natural scenario wherein transcriptional regulation is achieved when aTF competes against RNA polymerase for binding to a promoter in bacteria (*30*). The key here is that T4 DNA ligase reliably targets nicked DNA substrates to ligate the broken phosphodiester bond, but if the nicked TFBS is already occupied by an aTF, the T4 ligase cannot repair the nick. When the small-molecule analyte is present, the induced conformational change of aTF dissociates it from the nicked TFBS, thus allowing the nick to be “seamed” by T4 ligase. Critically, with this novel signal transduction system (aTF-NAST), it is only when the default nick in the DNA sequence is repaired that the broken sequence can be “rescued” as a suitable template for quantification using a variety of mature and robust DNA detection methods (Fig. 1B).

Considering that an enormously vast diversity of aTFs and attendant small-molecule effectors (analytes) have been identified in bacteria (*31*) and that this proposed aTF-NAST is amenable to any of the very mature and extremely robust DNA detection technologies that are used ubiquitously throughout life science and clinical medicine, we therefore proposed a general workflow for the development of small-molecule biosensors using aTF-NAST (Fig. 1C): First, specifically responding aTFs can be mined in bacteria or be obtained by engineering the recognition scope of a known aTF sensing analog of the analyte (*5*), and then, the proposed aTF-NAST is modularly deployed to construct biosensors by coupling with various standard DNA amplification–based detection methods, such as conveniently used RT-qPCR (*32*), RCA (*19*), RPA (*20*), and LAMP (*18*).

### Discovery of the interaction between aTF and nicked TFBS

**Fig. 2:**
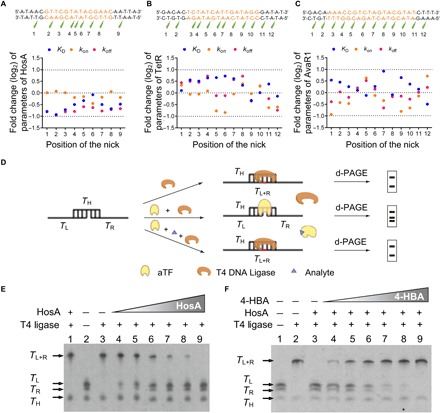
Confirmation of the aTF-NAST. (**A** to **C**) Variation of interaction parameters of HosA, TetR, and AvaR1 for their TFBSs with a nick at different positions, respectively. The TFBSs are indicated in orange. Each nick position is marked in green symbols. The interaction parameters (*K*_D_, *k*_on_, and *k*_off_) are fitted by built-in equations of a BLI machine (FortéBio) and listed in tables S2 to S4, respectively. Fold change values of *K*_D_, *k*_on_, and *k*_off_ were calculated relative to the intact one. (**D**) Principle for determination of the competitive binding between aTF and T4 DNA ligase on DNA containing nicked TFBS. Here, the DNA with a nick shows three bands, whereas “seamed” DNA shows two bands in d-PAGE. (**E**) Competition between HosA and T4 DNA ligase on DNA containing nicked TFBS of HosA. The concentration of T4 DNA ligase was 0.2 U/μl. Lanes 3 to 9: The concentrations of HosA were 0, 0.168, 0.335, 1.68, 3.35, 16.75, and 33.5 μM, respectively. (**F**) Examination of the nicked DNA freed by 4-HBA for T4 DNA ligase–mediated “seaming.” The concentrations of HosA and T4 ligase were 33.5 μM and 0.2 U/μl, respectively. Lanes 3 to 9: 0, 0.01, 0.05, 0.1, 0.5, 1, and 10 mM 4-HBA was added, respectively.

To our knowledge, there is no report about the interaction between bacterial aTF and its nicked TFBS. Thus, we first tested this hypothesis using a recombinant aTF HosA from *Escherichia coli* UMN026 (fig. S1A), which was known to specifically recognize the effector 4-HBA (*33*). We synthesized a 43–base pair (bp) DNA sequence containing 14-bp TFBS of HosA and then used an electrophoretic mobility shift assay (EMSA) to successfully confirm that the presence of 4-HBA caused the dissociation of HosA from this synthetic DNA sequence (fig. S2, A and B). Subsequently, the influence of a nick at various positions of TFBS on the binding of HosA was tested using biolayer interferometry (BLI) assay. The BLI sensorgrams showed that a nick positioned either within or close to (≤2 bp) the palindromic TFBS did not substantially alter the binding affinity of HosA for its TFBS (Fig. 2A and fig. S3): There were no notable differences in the equilibrium dissociation constant (*K*_D_), association constant (*k*_on_), or dissociation constant (*k*_off_) values between nicked and intact TFBSs (Fig. 2A and table S2). Further, to assess whether or not the binding of other aTFs is altered by the presence of a nick either within or adjacent to their TFBSs, we examined the interaction of another two randomly selected aTFs—TetR (fig. S1B) from *E. coli* (*34*) and AvaR1 (fig. S1C) from *Streptomyces avermitilis* (*35*, *36*)—with their respective nicked TFBSs. Both the BLI sensorgrams (figs. S4 and S5) and kinetic analyses (*K*_D_, *k*_on_, and *k*_off_; Fig. 2, B and C, and tables S3 and S4) showed no notable differences in the interactions of these two aTFs with their respective TFBSs. Thus, encouragingly, these three examples collectively illustrate that aTF is indeed able to interact with DNA containing nicked TFBS.

### Confirmation of the signal transduction process of aTF-NAST

To confirm the signal transduction process of our designed aTF-NAST, we used denaturing polyacrylamide gel electrophoresis (d-PAGE) to test the competitive binding of HosA and T4 DNA ligase to the DNA containing nicked TFBS (Fig. 2D). Specifically, we observed that the nick in this DNA sequence could be ligated by T4 DNA ligase when the binding competitor HosA was absent and also found that the amount of ligated DNA decreased with the increasing concentration of HosA (Fig. 2E), indicating that the binding of HosA indeed hinders the T4 DNA ligase–mediated ligation reaction. Further, whether the presence of 4-HBA freed the nicked TFBS for T4 DNA ligase–mediated ligation was also tested. We found that the amount of “seamed” DNA increased in a 4-HBA concentration–dependent manner (Fig. 2F). Thus, we conclude that our aTF-NAST can transduce the information perceived by the aTF (i.e., binding of 4-HBA) into an easy-to-detect DNA signal (the “rescued” DNA template).

Having demonstrated that our aTF-NAST works as designed, as a proof of concept, we next used aTF-NAST to develop small-molecule biosensors by coupling three widely used DNA detection methods: RT-qPCR (*32*), RCA (*19*), and RPA (*37*, *38*). The aTFs here that we selected are HosA (*33*), HucR (*14*), and TetR (*34*), specifically sensing the small molecules 4-HBA, UA, and TC, respectively.

### Development of biosensors by coupling aTF-NAST with RT-qPCR

**Fig. 3:**
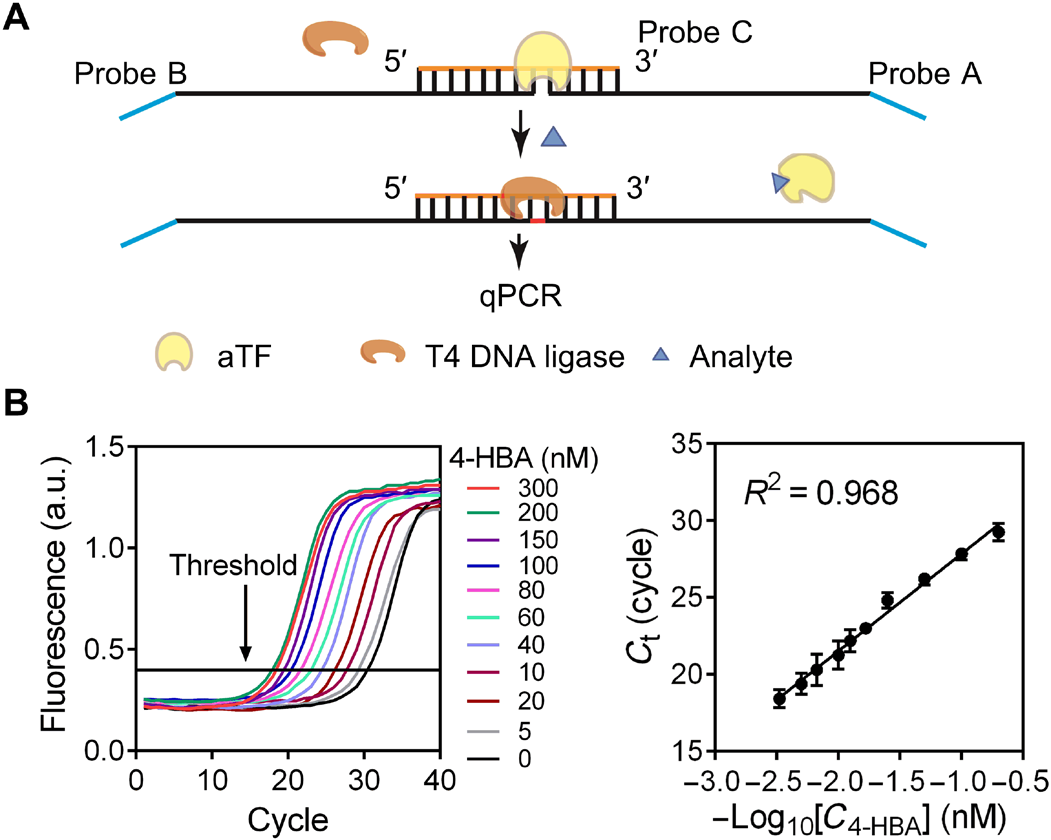
Development of biosensors by coupling aTF-NAST with RT-qPCR. (**A**) Schematic showing the strategy for combining aTF-NAST with RT-qPCR. (**B**) Amplification plots with different 4-HBA concentrations. a.u., arbitrary units. (**C**) Correlation analysis of the relationship between *C*_t_ and 4-HBA concentrations. Results shown are the averages of three independent experimental replicates with ±SD.

The schematic of how we coupled aTF-NAST with RT-qPCR to detect small molecules is shown in Fig. 3A. The template with a nicked TFBS of a given aTF is generated by annealing three single-stranded DNA sequences (probes A to C). When a small-molecule analyte is present, it binds to the aTF, resulting in the dissociation of the aTF from the nicked TFBS. Subsequently, probe A and probe B are ligated by T4 DNA ligase, and the ligated template is then quantified via RT-qPCR.

As a proof of concept, the construction of 4-HBA biosensor was implemented using well-characterized HosA and its nicked TFBS. To obtain the appropriate signal-to-noise (S/N) ratio of the biosensors, we first tested the amplification plots of RT-qPCR at different probe concentrations (fig. S6A). Twenty picomolar of probes A to C was chosen for further studies because it provided a significant change of the cycle threshold (*C*_t_) values compared to the background (S/N ratio > 3 and *P* < 0.001; fig. S6B). Then, the concentration of HosA was optimized. The *C*_t_ value generated by RT-qPCR showed a positive correlation with the concentration of HosA (fig. S6C), indicating that ligation reaction of the templates could be repressed by HosA. As 1 nM HosA met the criteria of both >90% change of the output signal and 10-fold above the apparent dissociation constant (*K*_d_) under the determined condition (fig. S6D), this concentration was adopted to construct the 4-HBA biosensor. In evaluating the performance of this 4-HBA biosensor, we observed the expected amplification plots outputted by RT-qPCR with different concentrations of 4-HBA (Fig. 3B) and obtained good correlation (*R*^*2*^ = 0.968) between the *C*_t_ values and the 4-HBA concentrations ranging from 5 to 300 nM (Fig. 3C); the limit of detection (LOD) was calculated to be 1.12 nM on the basis of the 3σ/slope rule (*39*).

### Development of biosensors by coupling aTF-NAST with RCA

**Fig. 4:**
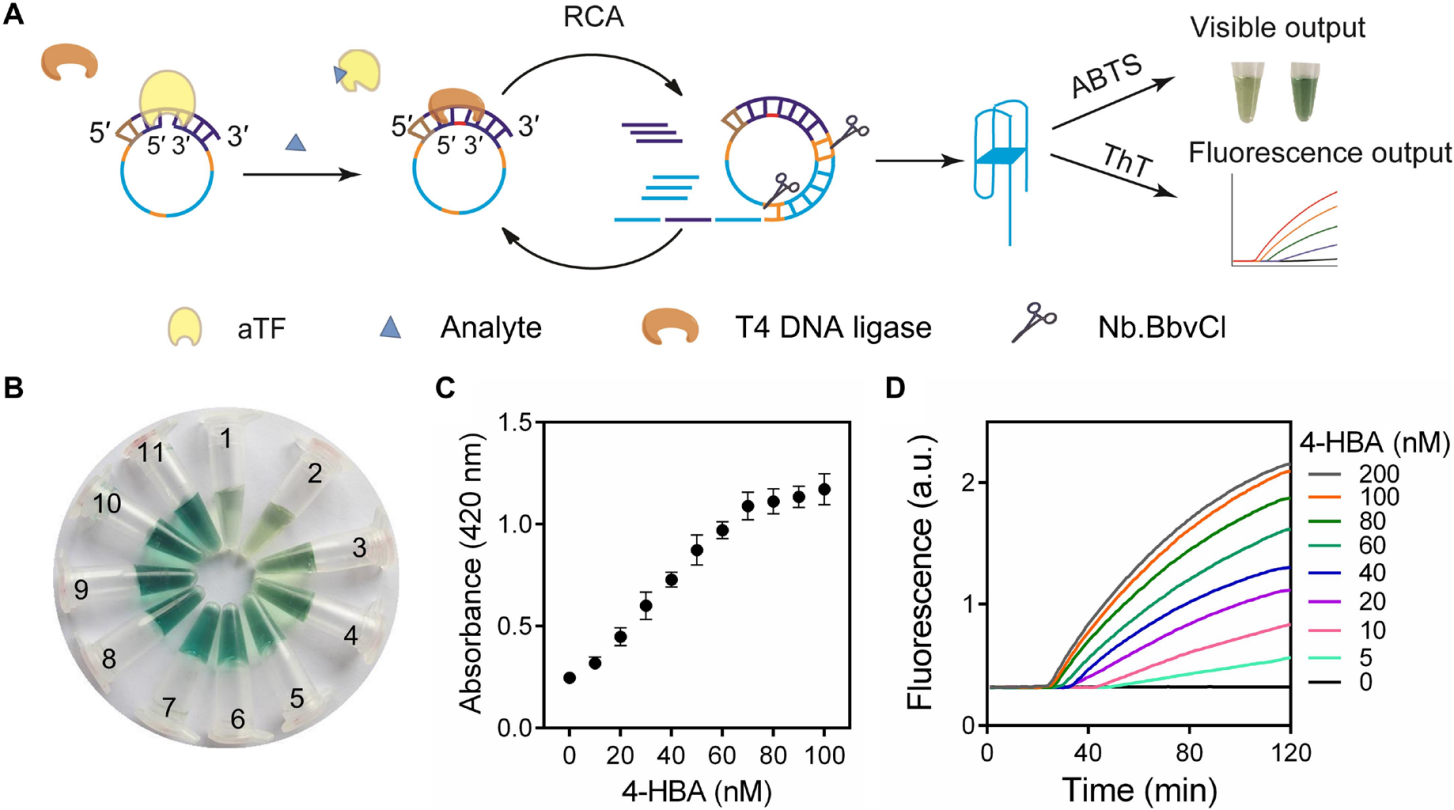
Development of biosensors by coupling aTF-NAST with RCA. (**A**) Schematic showing the strategy of combining aTF-NAST with RCA. In the template, orange marks recognition sites of Nb.BbvCI; blue marks the complementary sequences of G-quadruplex. (**B**) Visible output determined by peroxidase-mimicking G-quadruplexes. The concentrations of 4-HBA in tubes 1 to 11 were 0, 10, 20, 30, 40, 50, 60, 70, 80, 90, and 100 nM, respectively. (**C**) Quantification of the activity of peroxidase by colorimetric analysis. Results shown are the averages of three independent experimental replicates with ±SD. (**D**) Fluorescence-time curves for different concentrations of 4-HBA.

The schematic of coupling aTF-NAST with RCA to detect small molecules is presented in Fig. 4A. The linearized single-stranded DNA template included both terminal regions that can hybridize to the RCA primer, three nicking endonuclease Nb.BbvCI recognition sites, and two copies of the complementary sequence of a G-quadruplex (*40*). The RCA primer hybridizes to the linearized template, thereby forming a nicked TFBS that aTF can bind to; such a binding blocks T4 DNA ligase to circularize the template, thus preventing the RCA reaction (owing to the lack of an available circular template). When the small-molecule analyte is present, aTF is induced to dissociate from its TFBS, and the nicked template can be circularized by T4 DNA ligase. Then, an RCA reaction mediated by Phi29 DNA polymerase initiates the synthesis of a concatenated sequence copy using this circular template. Subsequently, nicking endonuclease Nb.BbvCI cuts the amplified concatenated sequence to generate G-quadruplexes and additional RCA primers, and the additional RCA primers, in turn, hybridizes to more linearized templates to trigger more T4 DNA ligase–mediated circularization and Phi29 DNA polymerase–mediated RCA reactions. Ultimately, plenty of G-quadruplexes are produced by multiple RCA reaction rounds. These G-quadruplexes can either serve as an efficient peroxidase-mimicking DNAzyme or output real-time fluorescence by interaction with thioflavin T (ThT) dye (*40*), thus enabling the developed biosensors with dual-output modes.

Another 4-HBA biosensor was constructed using the abovementioned strategy. Following the component optimization of this biosensor, amounts of 100 nM template and 50 pM primer were chosen for further studies because they provided a significant colorimetric change compared to the background (S/N ratio > 6 and *P* < 0.001; fig. S7, A and B). Subsequently, 3 nM HosA was adopted because this concentration was 10-fold above the apparent *K*_d_ and gives more than 90% reduction of RCA output signal under the determined RCA condition (fig. S7, C and D). Using the above optimized components, the biosensor was evaluated using visible peroxidase activity in a series of 4-HBA concentrations. To our delight, we found that a naked eye–detectable color change was achieved at a 4-HBA concentration of 10 nM (Fig. 4B). Further spectrophotometric analysis at 420 nm showed that the detection range for 4-HBA was 10 to 100 nM, and the LOD was calculated as 3.48 nM (Fig. 4C). Consistently, fluorescence generated by G-quadruplexes binding to ThT dye (*40*) was also monitored to analyze the performance of this biosensor, and we observed the nearly similar detection range (5 to 200 nM) and calculated the LOD (1.73 nM) (Fig. 4D).

### Development of biosensors by coupling aTF-NAST with RPA

**Fig. 5:**
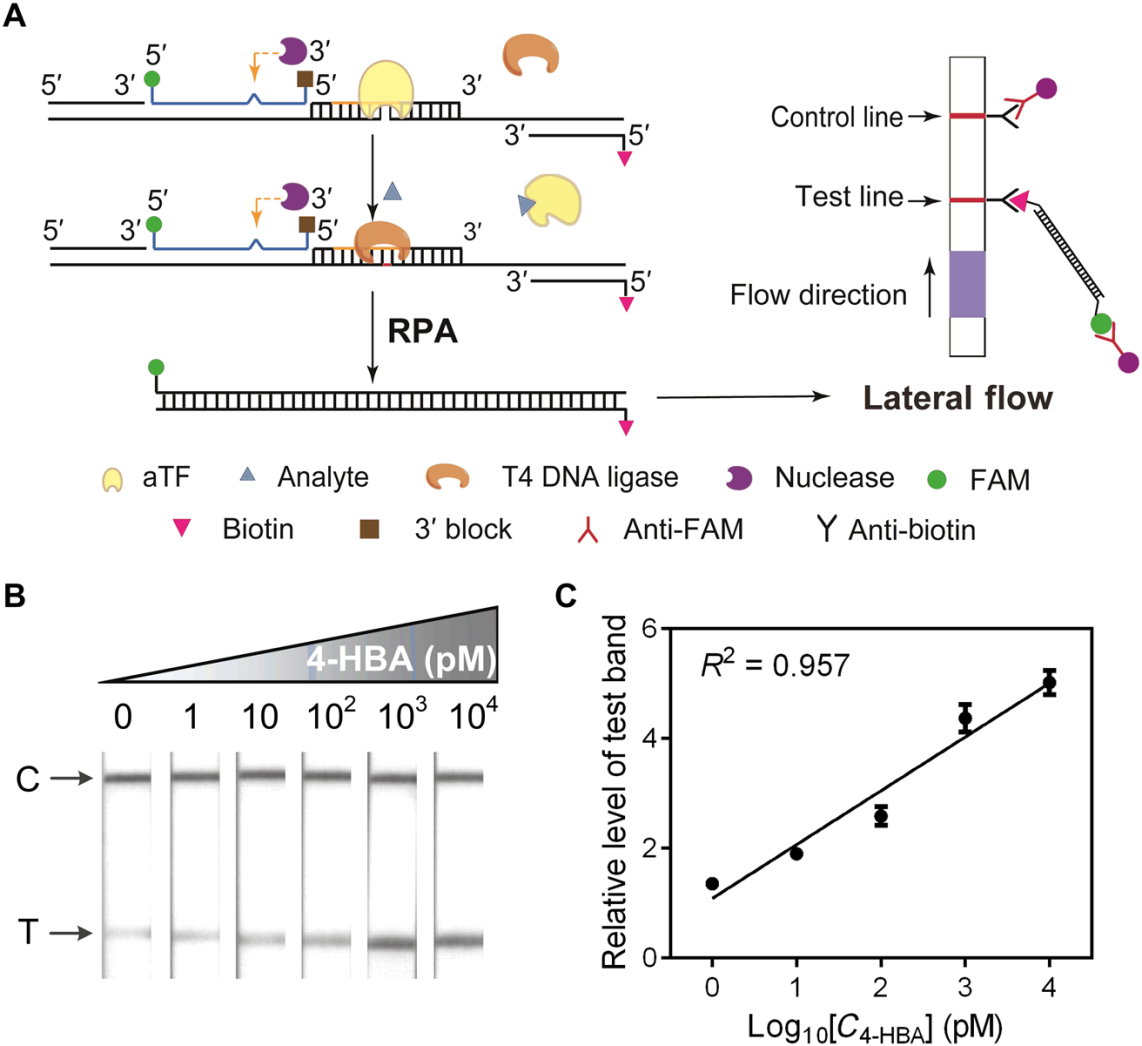
Development of biosensors by coupling aTF-NAST with RPA. (**A**) Schematic showing strategy of combining aTF-NAST with RPA. (**B**) LF strip–based detection of different 4-HBA concentrations. C and T indicate the control line and test line, respectively. (**C**) Calibration curve used to assess the relative intensity of the LF test line signals generated for different concentrations of 4-HBA. Results shown are the relative levels of the gray-scale intensity determined by ImageJ software. The gray scale of the LF test line without 4-HBA (control) was set to a value of 1. FAM, carboxyfluorescein-5-succimidyl ester.

RPA, which is famous for rapid and convenient nucleic acid detection with various outputs [e.g., the convenient lateral flow (LF) strips] (*37*, *38*), has been commercialized by TwistDx. We thus tried to use aTF-NAST to couple with this user-friendly technology to detect small molecules. A schematic of combining aTF-NAST and RPA with LF strip output is shown in Fig. 5A. Here, the template with nicked TFBS is generated by the same way as coupling aTF-NAST with RT-qPCR. The presence of the small-molecule analyte causes the dissociation of aTF from its nicked TFBS and thus enables T4 DNA ligase–mediated ligation of the nicked DNA template. Next, following RPA amplification step, the output signal is conveniently quantified by LF strips.

A 4-HBA biosensor was first implemented as an example. Similar to the abovementioned extensive optimization experiment, we obtained the optimal concentrations of the biosensor reagents: 5 fM template and 5 pM HosA (fig. S8, A to D). Then, the performance of this 4-HBA biosensor was evaluated by a series of 4-HBA concentrations. Results indicated that this biosensor was extremely sensitive: It could detect 4-HBA in concentrations as low as 1 pM (Fig. 5B). Its output (gray-scale intensity of test line read by a camera) could be quantified from 1 pM to 10 nM automatically using commonly available image processing software (Fig. 5C).

### Modularity of aTF-NAST for the development of small-molecule biosensors

**Fig. 6:**
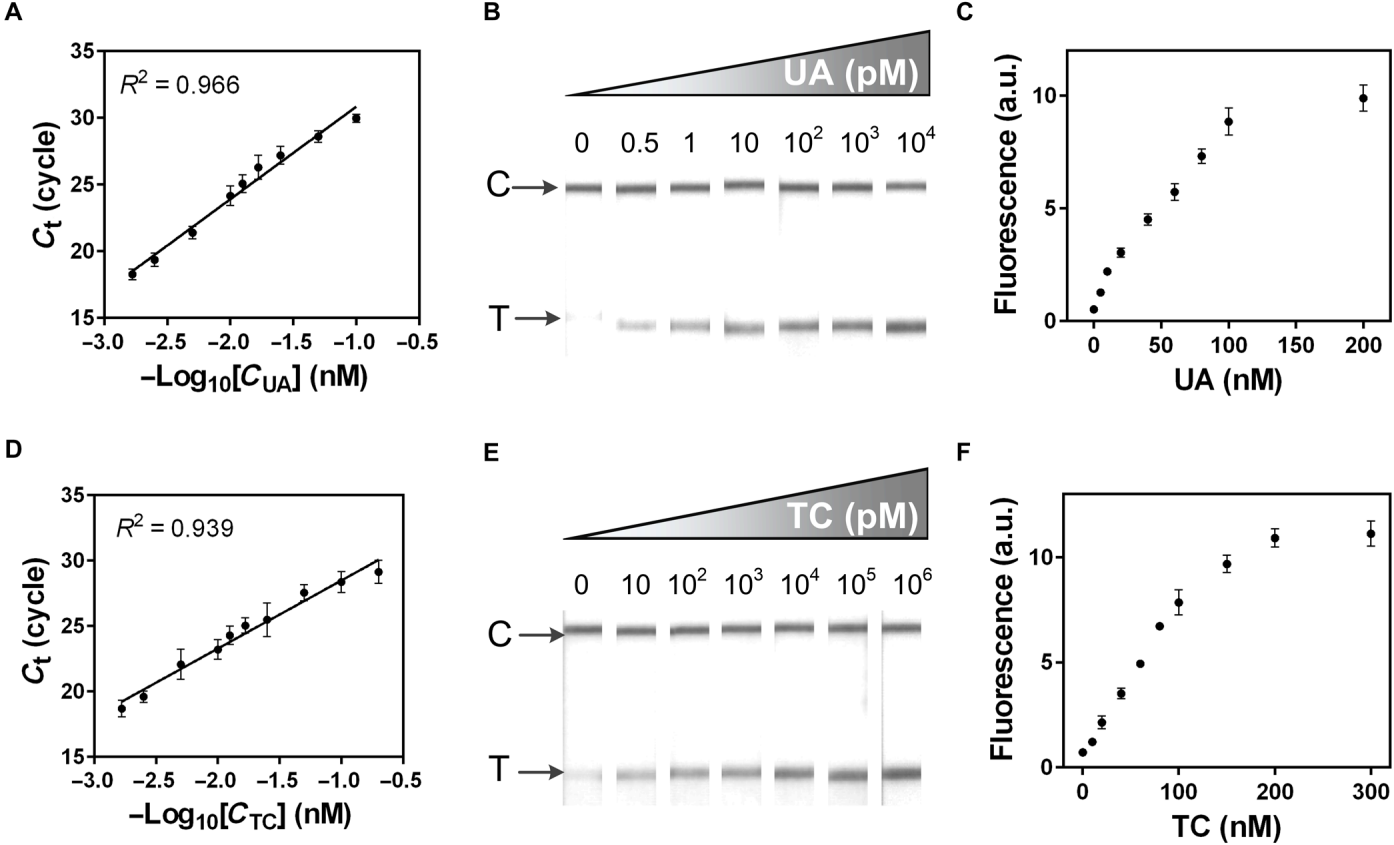
Development of UA and TC biosensors using aTF-NAST. (**A** to **C**) Performance of UA biosensors coupling HucR-NAST with, respectively, RT-qPCR, RPA, and RCA output. (**D** to **F**) Performance of TC biosensors coupling TetR-NAST with, respectively, RT-qPCR, RPA, and RCA output. For RCA output, here, we measured the fluorescence signal. In (**A**), (**C**), (**D**), and (**F**), the results shown are the averages of three independent experimental replicates with ±SD. In (**B**) and (**E**), the results shown are representative examples for multiple similar datasets.

Many aTFs have been identified to respond to diverse small molecules in bacteria (*5*, *31*). To demonstrate the broader applicability of our aTF-NAST, another two aTFs, HucR from *Deinococcus radiodurans* (*14*) and TetR from *E. coli* (*34*), were used to develop UA and TC biosensors, respectively. We first purified recombinant HucR and TetR to near homogeneity (fig. S1, B and D). Then, for each analyte, three biosensors were developed using the aTF-NAST to combine RT-qPCR (Fig. 6, A and D), RPA (Fig. 6, B and E), and RCA (Fig. 6, C and F), respectively. We were pleased to find that, when combining the same DNA detection method, the LOD and detection ranges of the developed biosensors were nearly the same order of magnitude (table S5), highlighting the modularity and the plug-and-play of our aTF-NAST that can be used with apparent ease for the construction of diverse aTF-based small-molecule biosensors.

### Performance of the biosensors for detecting real-life samples

Because of the lack of conventional biorecognition elements, to our knowledge, few of 4-HBA biosensors are available up to now. Thus, to evaluate the aTF-NAST–based biosensors, we benchmarked the performance of the developed UA and TC biosensors to the state-of-the-art ones constructed by other approaches. The results indicate that our biosensors belong to the most sensitive levels ever reported (tables S6 and S7). To investigate the validity of the developed biosensors to the detection of authentic samples, we spiked 4-HBA, UA, and TC into river water, human serum, and milk, respectively, and characterized the accuracy, precision, and recovery of the nine developed biosensors (table S8). The impressive precision (3.17 to 7.75%) indicates that our biosensors coupling aTF-NAST with mature DNA detection methods could ensure the robustness and stability for practical applications. Notably, the good accuracy (92.5 to 108.7%) and recovery (98.47 to 105.72%) of biosensors with RT-qPCR and RCA outputs indicate the great potential for quantification analysis. For biosensors coupling RPA with convenient LF strip output, the accuracy (86.8 to 116.1%) and recovery (83.68 to 118.43%) also comply with the criterion for fast and sensitive tests. Last, we compared the performance of the three UA biosensors with an automatic biochemistry analyzer for detection of UA in human serum in clinical diagnoses: Results further demonstrated the promising robustness and sensitivity of the aTF-NAST platform in real-life analyses (table S9). Notably, our biosensors with RCA or RPA output are convenient and independent of instruments. In addition to the demonstrable superiority of aTF-NAST–based biosensors, we would like to highlight the finding that the aTF-NAST platform provides a novel route for development of biosensors for in vitro detection of diverse small molecules, especially those that are undetectable by conventional recognition elements.

## DISCUSSION

Development of a novel biosensing workflow for a type of untapped recognition elements will genuinely infuse the detection field with new energy. Here, building on our new finding that aTFs could interact with their nicked TFBSs, we developed a robust and easy-to-implement signal transduction system, aTF-NAST, which could reliably and modularly transduce the signal of small molecules to an easily detectable DNA signal. Many mature and standard DNA detection methods therefore can be extended to quantify diverse small molecules via our aTF-NAST. We demonstrated both proof-of-concept examples (Figs. 3 to 5) and a variety of extensions (Fig. 6), highlighting the promise of aTF-NAST as a broader platform for a great many potential biosensing applications.

Numerous aTFs have been identified and characterized in bacteria (*5*, *31*). It has been well known that aTFs use the DBD to interact with the specific palindrome sequence (*29*). Here, we characterized an unprecedented capability of aTFs that they can bind to nicked TFBSs in vitro. Three aTFs (HosA, TetR, and AvaR1) were randomly selected to test the affinity for their TFBSs with a nick at different positions. To our surprise, we do not find that a nick within or adjacent the TFBS has a substantial effect on the binding activity of aTF. In contrast, some nick sites caused a slight increase of the affinity (decrease of *K*_D_ in Fig. 2, A to C). The underlying reason might be ascribed to the observation that a nick within or adjacent the TFBS increased the flexibility of this sequence, thus enabling the aTF to bind to this sequence more easily. Our finding extends the understanding of bacterial aTFs and broadens their potential applications in vitro.

The thermodynamic context of the design of the aTF-NAST biosensors is important for the technique to be generalizable (*41*). We set a general rule for the construction of biosensors. Since aTF-NAST could couple with diverse DNA amplification–based output approaches, to obtain the appropriate S/N ratio of the biosensors, we first determined the concentrations of nicked DNA template [figs. S6 (A and B), S7 (A and B), and S8 (A and B)]. Then, to ensure the accurate measurability of the biosensors, the appropriate concentrations of aTFs that we chose must meet the criteria of both 10-fold above the apparent *K*_d_ and >90% change of the output signal under the determined condition [figs. S6 (C and D), S7 (C and D), and S8 (C and D)]. Taking the 4-HBA biosensor with RT-qPCR output as an example, the apparent *K*_d_ of HosA is 0.09 nM and the HosA concentration that we chose is 1 nM; such a concentration results in 91% inhibition of the output signal (fig. S6D). This rule ensures that our nine biosensors coupling aTF-NAST with three standard and mature DNA detection methods (i.e., RT-qPCR, RCA, and RPA) all have stable outputs (tables S8 and S9). In addition, we also checked the thermodynamic of the interaction between aTFs and the corresponding small molecules. TC binding to TetR has been used as a paradigm, and the affinity has been well characterized as ~10^−8^ M (*34*). We further characterized the *K*_D_ values of HucR and HosA for their respective analytes UA and 4-HBA using isothermal titration calorimetry (ITC) (fig. S9, A and B). The *K*_D_ values of HucR and HosA are 9.35 ± 0.99 μM and 4.74 ± 0.25 μM, respectively. All the detection ranges of our developed biosensors were lower than 10-fold of the corresponding *K*_D_ of aTF-binding small molecules. Therefore, it stands to reason that the response of aTF to a small molecule shows a concentration-dependent manner and that the biosensors give the good results with linear response rates. Notably, the affinity of T4 DNA ligase for nicked DNA is 0.025 ± 0.001 μM (*42*) (fitted by both binding steps in the two-step binding mode, it is consistent with the *K*_m_ determined for this substrate in turnover experiments). When the allosteric signal is presented, the affinity of aTF for DNA is usually reduced by several orders of magnitude. Therefore, in our aTF-NAST, T4 DNA ligase is qualified to compete to bind the nicked DNA when the aTF interacts with small molecules.

It is well known that bacterial aTFs have been extensively used for biosensing genetic switches in vivo (*5*–*12*). However, how to further exploit and use the potential of small-molecule recognition of aTF in vitro is seldom investigated. Here, using the newly discovered capability of aTF binding to nicked TFBS, we demonstrated a reliable and modular aTF-NAST to transduce small-molecule recognition of aTFs to easily detectable DNA signal in homogeneous buffers in vitro. We anticipate that our aTF-NAST as a platform will be a starting point for more extended biosensing applications. There are three reasons to support our claim: (i) Numerous aTFs sensing diverse small molecules have been identified in bacteria (*31*), which are an untapped mine for in vitro biosensing. In addition, aTFs can also be engineered to respond to new ligands (analyte) (*5*). Thus, the aTF-NAST gives us a chance to develop biosensors for the in vitro detection of diverse small molecules, especially those that are unable to be sensed by conventional recognition elements (such as enzymes, antibodies, and aptamers). (ii) Our aTF-NAST shows perfect compatibility with many of the very mature and extremely robust DNA detection methods [e.g., combining commercialized LAMP kit (*18*) and just published CRISPR-Cas–mediated supersensitive nucleic acid detection method (*21*–*24*), etc.], not limited to the three DNA detection methods in the present work. The compatibility with standard and mature DNA detection methods ensures the good robustness and reproducibility of the developed biosensors (table S8). In addition, the ligated DNA signal is also amendable to many optical or electrical detection methods, which is also promising in the future. (iii) The aTF-NAST is ready to use. The modularity of our aTF-NAST can speed up the development of diverse small-molecule biosensors with urgent needs. Moreover, the whole signal transduction process of aTF-NAST is performed in a homogeneous buffer in vitro. In summary, taking advantage of those enormous aTFs that have been identified to sense diverse small molecules (*31*), our developed aTF-NAST indeed opens a novel route to develop small-molecule biosensors.

## MATERIALS AND METHODS

### Reagents

Oligonucleotides used in this work (table S1) were synthesized and high-performance liquid chromatography–purified by Sangon Biotechnology Co., Ltd. T4 DNA ligase, Phi29 DNA polymerase, nicking endonuclease Nb.BbvCI, and deoxynucleotide triphosphates (dNTPs) were obtained from New England BioLabs Inc. FastFire qPCR PreMix (SYBR Green) was obtained from Tiangen Biotech Co., Ltd. (China). Hemin, ThT, dimethysulfoxide (DMSO), hydrogen peroxide (H_2_O_2_), 2,2′-azino-bis(3-ethylbenzothiozoline-6-sulfonicacid) diammonium salt (ABTS^2−^), and bovine serum albumin (BSA) were purchased from Sigma-Aldrich. Ultrapure water obtained from a Millipore filtration system was used throughout. All other chemical reagents used in this work were of analytical grade without further purification. The buffer solutions used were as follows: TE buffer [10 mM tris-HCl, 0.1 mM EDTA, and 0.1 M NaCl (pH 7.8)], KT buffer [100 mM MES, 50 mM tris-HCl, 40 mM KCl, 0.05% Triton X-100, and 1% DMSO (pH 6.2)], HBS-EP buffer [10 mM Hepes, 15 mM NaCl, 3 mM EDTA, 0.005% Tween 20, and 0.1% BSA (pH 7.4)], 5× TBE buffer [446 mM tris, 446 mM boric acid, and 10 mM EDTA (pH 8.3)], and 5× d-PAGE loading buffer [1 M NaOH (1 ml), formamide (95 ml), and bromophenol blue (0.05 g)]. The oligonucleotide stock solutions (10 μM) were prepared with 10 mM TE buffer and kept frozen. A stock solution of hemin (100 nM) was prepared with DMSO and stored at −20°C in the dark. All other chemical reagents used in this work were of analytical grade without further purification.

### Preparation of recombinant aTFs

The sequences of *hosA* (*33*), *avaR1* (*35*), *hucR* (*14*), and *tetR* (*34*) were synthesized and cloned into the Nde I/Xho I sites of pET23b. Expression and purification of these aTFs were performed as previously described (*14*).

### Electrophoretic mobility shift assay

Experiment conditions for EMSAs and data recording were same as described previously (*13*). Probe DNA_TFBS_ was obtained by annealing E1 and E2 (table S1). DNA_TFBS_ (8.55 nM) with different amounts (ranging from 0 to 1540 nM) of purified HosA were mixed in a binding buffer [20 mM tris-HCl, 2 mM dithiothreitol, 5 mM MgCl_2_, BSA (0.5 mg/ml), and 5% glycerol (pH 7.5)] at a total volume of 20 μl, followed by incubation at 25°C for 20 min. To further test whether HosA can be dissociated from DNA_TFBS_ by 4-HBA, different amounts (ranging from 0 to 40 mM) of 4-HBA was added and incubated together with 8.55 nM DNA_TFBS_ and 154 nM HosA. After incubation, the binding mixture was loaded onto a native gel (4% polyacrylamide).

### BLI assay

The 5′-terminal end of the forward primer DNA_TFBS_-F0 was labeled by biotin (table S1). The biotin-labeled DNA sequences containing intact TFBS of HosA (DNA_HosA_-0) were generated by annealed primers (DNA_HosA_-F0 and DNA_HosA_-R0). The biotin-labeled DNA sequences containing nicked TFBS of HosA (DNA_HosA_-N, where N indicates the nick position) were obtained by annealed primers (DNA_HosA_-F0, DNA_HosA_-RN-1, and DNA_HosA_-RN-2; table S1). Other biotin-labeled DNA sequences containing intact or nicked TFBS of TetR and AvaR1 were obtained in the same manner as for that of HosA. The primer annealing was performed by heating at 95°C for 5 min, followed by slowly cooling to room temperature. The generated bio-DNA_TFBS_ were verified by agarose gel electrophoresis and were quantified using a NanoVue plus Spectrophotometer (GE Healthcare). Kinetics between aTFs (HosA, TetR, and AvaR1) and their nicked TFBSs were determined by BLI assay using an Octet RED96 system (FortéBio). Briefly, the process consisted of five steps: balance, DNA loading, rebalance, association, and dissociation. The blank tests were carried out by using HBS-EP buffer instead of aTFs in the association step and used for baseline correction. *k*_on_, *k*_off_, and *K*_D_ (*K*_D_ = *k*_off_/*k*_on_) values were calculated by fitting the processed data (baseline correction and normalization) with a 1:1 model using the Octet Analysis System 21 CFR Part 11 (version 9.0). The regression coefficients (*R*^*2*^ value) and error values were used to assess the quality of the fits to the data.

### ITC assay

MicroCal iTC200 was used for the characterization of the interaction between aTFs and small molecules. HucR and HosA were diluted in an ITC buffer [150 mM NaCl and 50 mM tris-HCl (pH 7.5)] with final concentrations of 25 and 20 μM, respectively. The small molecules UA and 4-HBA were dissolved in the same ITC buffer with a final concentration of 250 μM. The ITC conditions were as follows: 330 μl of the aTF was first injected in the sample cell. Then, the titration was initiated with a first injection of 0.4 μl of the corresponding small molecule, followed by 19 injections of 2 μl at 25°C. The blank tests were carried out by using an ITC buffer instead of aTFs in the titration step and were used for baseline correction. The instrument software was used to calculate the normalized heats released from each injection.

### Confirmation of the signal transduction process of aTF-NAST

The competitive interaction between HosA and T4 DNA ligase with the nicked TFBS of HosA was examined using d-PAGE. *T*_L_, *T*_R_, and *T*_H_ with a ratio of 1:1:1 were annealed to form the DNA template. Different concentrations of HosA (0, 0.168, 0.335, 1.68, 3.35, 16.75, and 33.5 μM) and 200 nM DNA template were then incubated in 20 μl of 1× T4 DNA ligase buffer at 25°C for 20 min to allow complete interaction between protein and DNA. After that, 4 U of T4 DNA ligase was added to the DNA-HosA complex with an incubation period of 30 min at 37°C. Then, the reaction was terminated by heating at 85°C for 10 min and was immediately cooled on the ice. The complexes were then mixed with 1× d-PAGE loading buffer. For d-PAGE assay, the samples were loaded on a 15% d-PAGE gel [volume (5 ml): 15% acrylamide (acrylamide and bis-acrylamide at a ratio of 29:1), 5× TBE (1 ml), tetramethylethylenediamine (TEMED; 2.5 μl), and 10% ammonium persulfate (25 μl), and 7 M urea]. The electrophoresis was run at a constant current of 5 mA for about 20 min, using 1× TBE as running buffer. The gels were stained with SYBR Gold for 40 min and then photographed under a Transilluminator.

### Coupling aTF-NAST with RT-qPCR

To optimize the concentration of probes, the annealed template consisted of equal proportions of HBA–probe A, HBA–probe B, and HBA–probe C (table S1) that were diluted from 20 fM to 20 nM, 1× T4 DNA ligase buffer, and 1 U T4 DNA ligase in a reaction volume of 10 μl. The ligation reaction mixture was incubated at 37°C for 30 min to complete the ligation reaction. After the inactivation of T4 DNA ligase at 85°C for 10 min, the mixture was slowly cooled to room temperature. For RT-qPCR detection, the final volume of reaction was 20 μl including 10 μl of 2× FastFire qPCR PreMix, 2 μl of ligation products, and 0.2 μM forward and reverse primers. The RT-qPCR reaction was performed on a Roche LightCycler 480II real-time PCR system by using a hot start of 95°C for 1 min, followed by 40 cycles of 95°, 55°, and 72°C for 5, 10, and 15 s, respectively. The real-time fluorescence intensity was simultaneously monitored after elongation of each cycle. The concentration of probes used to construct each biosensor was optimized to a satisfied S/N ratio of >3. The S/N ratio was calculated as Δ*C*_t_/(Δ*C*_tmin_). Δ*C*_t_ indicated the difference value between the *C*_t_ value of nontemplate control and *C*_t_ value of samples. Δ*C*_tmin_ indicated the minimum Δ*C*_t_. To optimize the concentration of HosA, 10 μl of mixture containing 20 pM annealed templates, different amounts of HosA from 0 pM to 10 nM, and 1× T4 DNA ligase buffer was incubated at 25°C for 20 min to allow HosA binding to the annealed template. Then, 1 U of T4 DNA ligase was added, and the ligation reaction mixture was incubated at 37°C for 30 min. The RT-qPCR reaction was conducted as above. To characterize the 4-HBA biosensor with RT-qPCR output, 10 μl of ligation reaction mixture consisting of 20 pM annealed templates, 1 nM HosA, different amounts of 4-HBA (from 0 to 300 nM), and 1× T4 DNA ligase buffer was incubated at 25°C for 20 min. Then, the ligation and RT-qPCR reactions were conducted as above. For the construction of the TC and UA biosensors, procedures were the same as the abovementioned optimization of the 4-HBA biosensor. For novice users, we recommend following the instructions of the RT-qPCR kit and instrument exactly provided by the producers.

### Coupling aTF-NAST with RCA

To determine the optimal concentration of the primer, 100 nM circle template (HBA-CT) and different concentrations of RCA primer (HBA-P) (table S1) were annealed in 1× T4 DNA ligase buffer to form template-primer complexes with a nicked DNA_TFBS_. Then, 2 U of T4 DNA ligase was added, and the mixture was incubated at 37°C for 30 min to seal the 5′-phophate and 3′-hydroxyl ends of the nicked circular template. For RCA reaction, 2 μl of Phi29 DNA polymerase buffer, 0.5 μl of 25 mM dNTPs, 0.1 μl of phi29 DNA polymerase (100 U/μl), and 1 μl of Nb.BbVCI (10 U/μl) were introduced into the system at a final volume of 20 μl. The RCA reaction mixture was incubated at 37°C for 2 hours on a PCR thermocycle instrument, and the reaction was terminated by heating at 85°C for 10 min. To determine the optimal HosA concentration, 100 nM HosA-circle probe and 50 pM HosA-PC were used to prepare the template-primer complexes. Then, different concentrations of HosA (0, 1.5, 3, 4.5, and 6 nM) were mixed with the template-primer complexes and incubated at 25°C for 20 min to allow complete interaction between protein and DNA. Subsequently, the ligation and RCA reaction were performed as mentioned above.

To characterize the 4-HBA biosensor with RCA output, different concentrations of the 4-HBA from 0 to 200 nM were added to the aTFs-DNA complex under the above determined conditions and incubated at 37°C for 30 min. The ligation and RCA reactions were performed as above. Then, visible output and real-time fluorescence output were measured. For visible output, the solutions of 3.6 mM ABTS^2−^ and 3.6 mM H_2_O_2_ were simultaneously added to the DNAzyme solution and diluted with TE buffer to yield a total volume of 100 μl. Data were obtained by recording the absorbance at 420 nm after 5 min of the reaction, and photographs of the results were taken by the Tanon 1600 Gel Imaging System. For real-time fluorescence output, the RCA reaction containing 5 μM ThT was incubated at 37°C on a Roche LightCycler 480II real-time PCR system, and real-time fluorescence was monitored at intervals of 1 min. For the construction of TC and UA biosensors, the procedures were the same as above for the optimization of the 4-HBA biosensor. For the construction of the UA and TC biosensors, the procedures were the same as the abovementioned optimization of the 4-HBA biosensor.

### Coupling aTF-NAST with RPA

A 50 μl of reaction containing 420 nM RPA primers (RPA-P1 and RPA-P2), 120 nM RPA-probe (table S1), and 1× rehydration buffer was performed following the manual of a TwistAmp Basic kit (no. TANFO02KIT, TwistDx, UK). To determine the optimal concentration of the DNA template, different concentrations (0.1, 0.5, 5, 50, and 500 fM and 5 and 50 pM) of three oligonucleotides (HBA-A, HBA-B, and HBA-C; table S1) were used to prepare templates by annealing. Then, 2 U of T4 DNA ligase was added, and the mixture was incubated at 37°C for 30 min. Subsequently, RPA reaction followed by convenient LF strip detection was performed. To determine the optimal HosA concentration, different concentrations of HosA (0.5, 1, 2, 3, 4, and 5 pM) were mixed with DNA duplexes and incubated at 25°C for 20 min to allow complete interaction between protein and DNA. Then, ligation and detection reactions were carried out as above. To characterize the 4-HBA biosensor with RPA output, 10 μl of reaction mixture consisting of 5 fM annealed template, 5 pM HosA, different amounts of 4-HBA (from 0 to 10 nM), and 1× T4 DNA ligase buffer was incubated at 25°C for 20 min. Then, the ligation and RPA reaction were performed as above. For the construction of TC and UA biosensors, the procedures were the same as above for the optimization of the 4-HBA biosensor. For novice users, following the instructions of RPA kit exactly is critical, which could ensure that sample cross-contamination is avoided.

## Supplementary Material

http://advances.sciencemag.org/cgi/content/full/4/11/eaau4602/DC1

## SUPPLEMENTARY MATERIALS

Supplementary material for this article is available at http://advances.sciencemag.org/cgi/content/full/4/11/eaau4602/DC1 Fig. S1. Purified recombinant aTFs examined by SDS-PAGE. Fig. S2. Verification of the activity of purified HosA by EMSA. Fig. S3. Interaction kinetics of HosA and its intact or nicked TFBSs. Fig. S4. Interaction kinetics of TetR and its intact or nicked TFBSs. Fig. S5. Interaction kinetics of AvaR1 and its intact or nicked TFBSs. Fig. S6. Optimizing the system for combining aTF-NAST with RT-qPCR. Fig. S7. Optimizing the system for combining aTF-NAST with RCA. Fig. S8. Optimizing the system for combining aTF-NAST with RPA. Fig. S9. Interaction between aTFs and corresponding small molecules determined by ITC. Table S1. Primers and oligonucleotides used in this work. Table S2. Parameters of the interaction dynamics between HosA and intact or nicked TFBSs. Table S3. Parameters of the interaction dynamics between TetR and intact or nicked TFBSs. Table S4. Parameters of the interaction dynamics between AvaR1 and intact or nicked TFBSs. Table S5. Performance of the developed aTF-based biosensors in this study. Table S6. Comparison with previously reported UA biosensors. Table S7. Comparison with previously reported TC biosensors. Table S8. Performance of the developed biosensors. Table S9. Comparison of the performance of the developed UA biosensors in a clinical test. References (*43*–*75*)
