# Supplementary material for: Harnessing a previously unidentified capability of bacterial allosteric transcription factors for sensing diverse small molecules in vitro
Source: Sci Adv. 2018 Nov 28;4(11):eaau4602. doi: 10.1126/sciadv.aau4602 (PMC6261655; doi:10.1126/sciadv.aau4602)
Supplement: http://advances.sciencemag.org/cgi/content/full/4/11/eaau4602/DC1 [file supp_4_11_eaau4602__index.html]

Science Advances | Science Advances

## Supplementary Materials

**This PDF file includes:**

- Fig. S1. Purified recombinant aTFs examined by SDS-PAGE.
- Fig. S2. Verification of the activity of purified HosA by EMSA.
- Fig. S3. Interaction kinetics of HosA and its intact or nicked TFBSs.
- Fig. S4. Interaction kinetics of TetR and its intact or nicked TFBSs.
- Fig. S5. Interaction kinetics of AvaR1 and its intact or nicked TFBSs.
- Fig. S6. Optimizing the system for combining aTF-NAST with RT-qPCR.
- Fig. S7. Optimizing the system for combining aTF-NAST with RCA.
- Fig. S8. Optimizing the system for combining aTF-NAST with RPA.
- Fig. S9. Interaction between aTFs and corresponding small molecules determined by ITC.
- Table S1. Primers and oligonucleotides used in this work.
- Table S2. Parameters of the interaction dynamics between HosA and intact or nicked TFBSs.
- Table S3. Parameters of the interaction dynamics between TetR and intact or nicked TFBSs.
- Table S4. Parameters of the interaction dynamics between AvaR1 and intact or nicked TFBSs.
- Table S5. Performance of the developed aTF-based biosensors in this study.
- Table S6. Comparison with previously reported UA biosensors.
- Table S7. Comparison with previously reported TC biosensors.
- Table S8. Performance of the developed biosensors.
- Table S9. Comparison of the performance of the developed UA biosensors in a clinical test.
- References (*43*–*75*)

Download PDF

**Files in this Data Supplement:**

- Adobe PDF - aau4602\_SM.pdf
