# Supplementary material for: Harnessing a previously unidentified capability of bacterial allosteric transcription factors for sensing diverse small molecules in vitro
Source: Sci Adv. 2018 Nov 28;4(11):eaau4602. doi: 10.1126/sciadv.aau4602 (PMC6261655; doi:10.1126/sciadv.aau4602)
Supplement: http://advances.sciencemag.org/cgi/content/full/4/11/eaau4602/DC1 [file aau4602_SM.pdf]

Science Advances
AAAS

advances.sciencemag.org/cgi/content/full/4/11/eaau4602/DC1

Supplementary Materials for

Harnessing a previously unidentified capability of bacterial allosteric transcription factors for sensing diverse small molecules in vitro

Jiaqian Cao, Yongpeng Yao, Keqiang Fan, Gaoyi Tan, Wensheng Xiang, Xuekui Xia,
Shanshan Li*, Weishan Wang*, Lixin Zhang*

*Corresponding author. Email: lxzhang@ecust.edu.cn (L.Z.); wangws@im.ac.cn (W.W.); ssli@ippcaas.cn (S.L.)

Published 28 November 2018, *Sci. Adv.* **4**, eaau4602 (2018)
DOI: 10.1126/sciadv.aau4602

This PDF file includes:

Fig. S1. Purified recombinant aTFs examined by SDS-PAGE.
Fig. S2. Verification of the activity of purified HosA by EMSA.
Fig. S3. Interaction kinetics of HosA and its intact or nicked TFBSs.
Fig. S4. Interaction kinetics of TetR and its intact or nicked TFBSs.
Fig. S5. Interaction kinetics of AvaR1 and its intact or nicked TFBSs.
Fig. S6. Optimizing the system for combining aTF-NAST with RT-qPCR.
Fig. S7. Optimizing the system for combining aTF-NAST with RCA.
Fig. S8. Optimizing the system for combining aTF-NAST with RPA.
Fig. S9. Interaction between aTFs and corresponding small molecules determined by ITC.
Table S1. Primers and oligonucleotides used in this work.
Table S2. Parameters of the interaction dynamics between HosA and intact or nicked TFBSs.
Table S3. Parameters of the interaction dynamics between TetR and intact or nicked TFBSs.
Table S4. Parameters of the interaction dynamics between AvaR1 and intact or nicked TFBSs.
Table S5. Performance of the developed aTF-based biosensors in this study.
Table S6. Comparison with previously reported UA biosensors.
Table S7. Comparison with previously reported TC biosensors.
Table S8. Performance of the developed biosensors.
Table S9. Comparison of the performance of the developed UA biosensors in a clinical test.
References (*43–75*)

Supplementary Figures

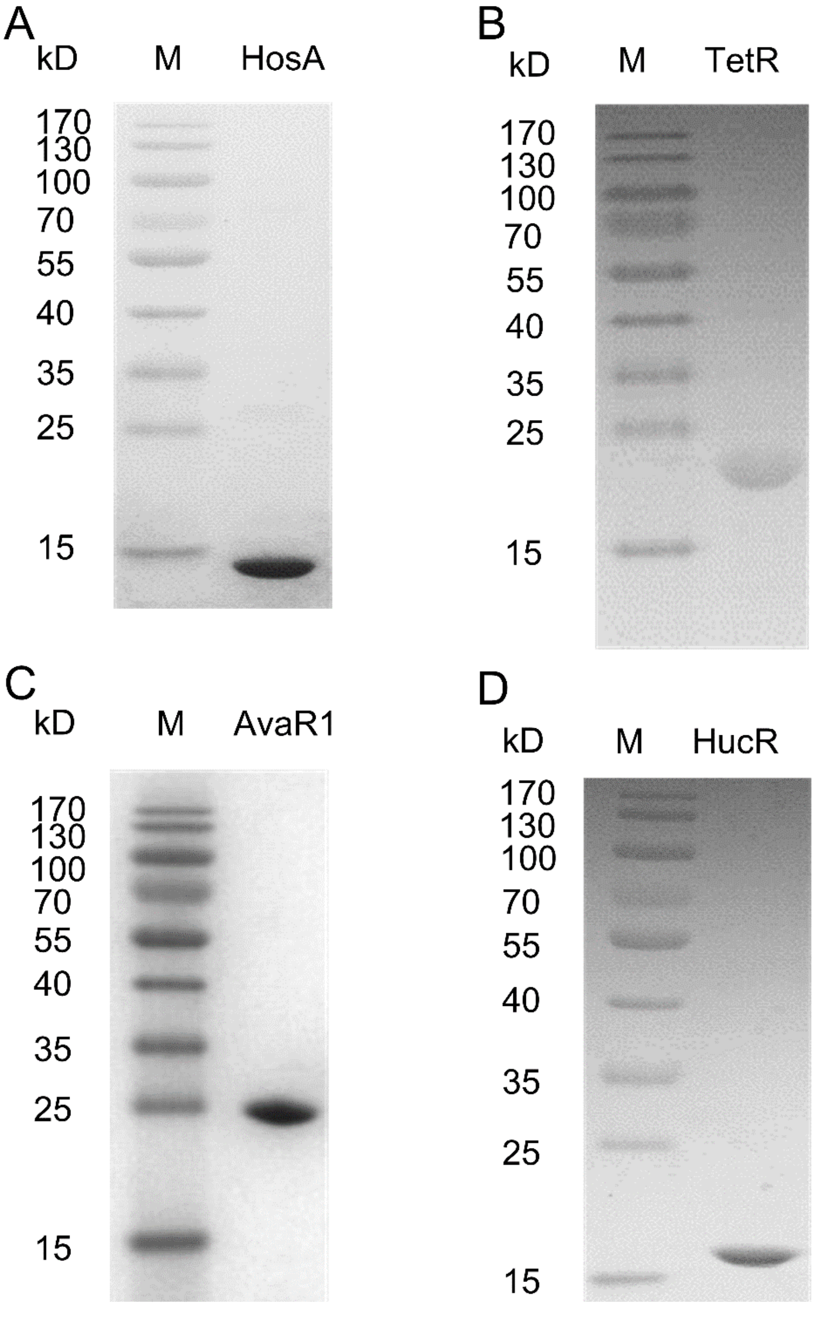

Fig. S1. Purified recombinant aTFs examined by SDS-PAGE. (**A**) SDS-PAGE result of purified HosA. (**B**) SDS-PAGE result of purified TetR. (**C**) SDS-PAGE result of purified AvaR1. (**D**) SDS-PAGE result of purified HucR. Lane M, protein marker (15-170 KDa).

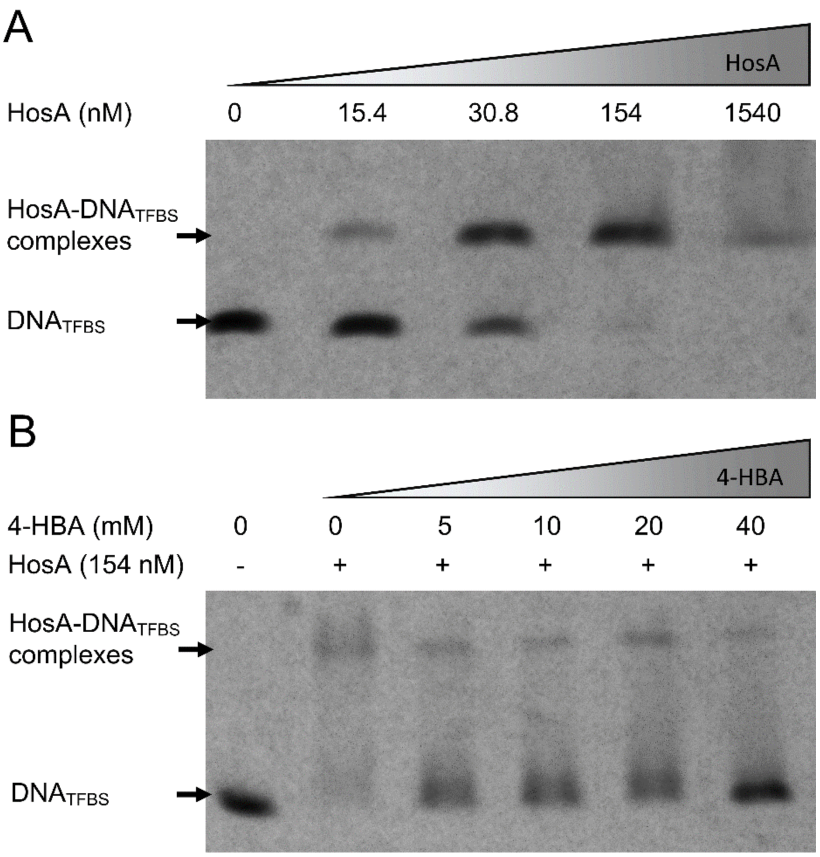

Fig. S2. Verification of the activity of purified HosA by EMSA. (**A**) Interaction between HosA and DNA containing TFBS (DNA_{TFBS}). 8.55 nM of DNA_{TFBS} was used. (**B**) The dissociation of HosA-DNA_{TFBS} complexes by different concentrations of 4-HBA. 8.55 nM of DNA_{TFBS} and 154 nM of HosA were used.

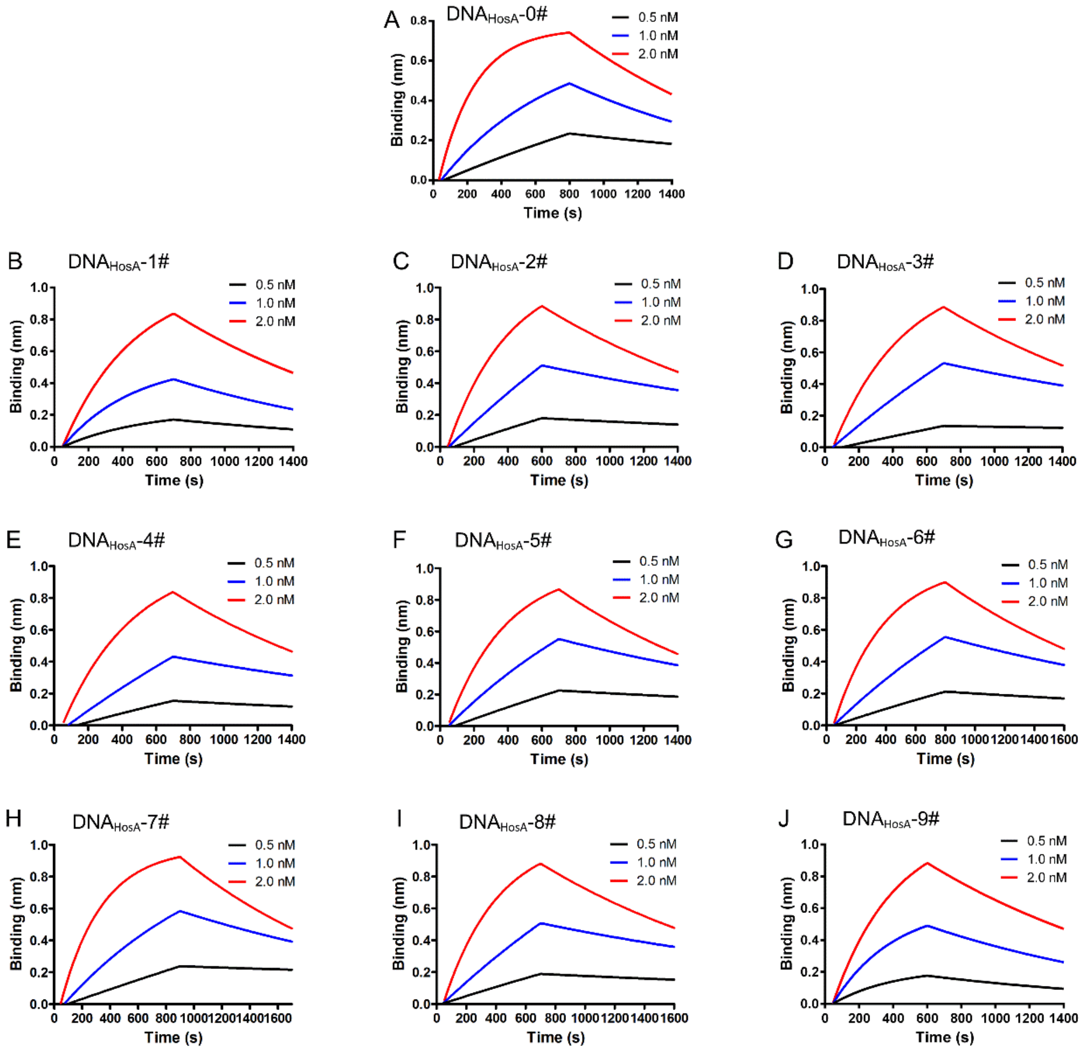

Fig. S3. Interaction kinetics of HosA and its intact or nicked TFBSs. (**A**) Sensorgram of the interaction between HosA and intact TFBS (DNA$_{HosA}$-0) determined by BLI. (**B**), (**C**), (**D**), (**E**), (**F**), (**G**), (**H**), (**I**) and (**J**) show sensorgrams of the interaction between HosA and its TFBSs with a nick at various positions (DNA$_{HosA}$-N, N indicates nicked position in Fig. 2A). 0.8 mM of DNA$_{HosA}$-N was used for loading step. The values of K_D, k_{on} and k_{off} are listed in table S2.

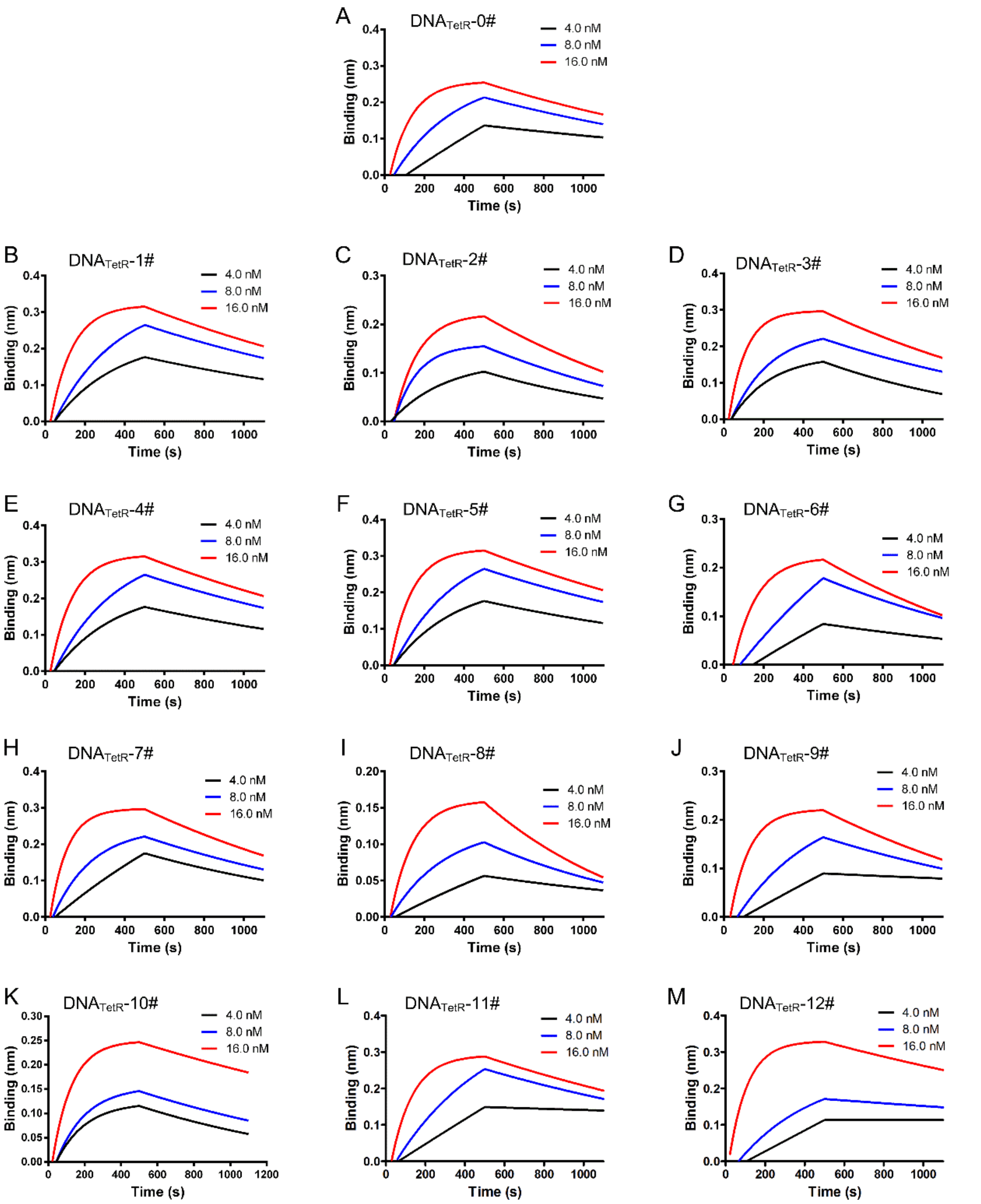

Fig. S4. Interaction kinetics of TetR and its intact or nicked TFBSs. (**A**) Sensorgram of the interaction between TetR and DNA containing intact TFBS (DNA_{TetR}-0) determined by BLI. (**B**), (**C**), (**D**), (**E**), (**F**), (**G**), (**H**), (**I**), (**J**), (**K**), (**L**) and (**M**) show sensorgrams of the interaction between TetR and its TFBSs with a nick at various positions (DNA_{TetR}-N, N indicates nicked position in Fig. 2B). 0.8 mM of DNA_{tetR}-N was used for loading step. The values of K_D, k_{on} and k_{off} are listed in table S3.

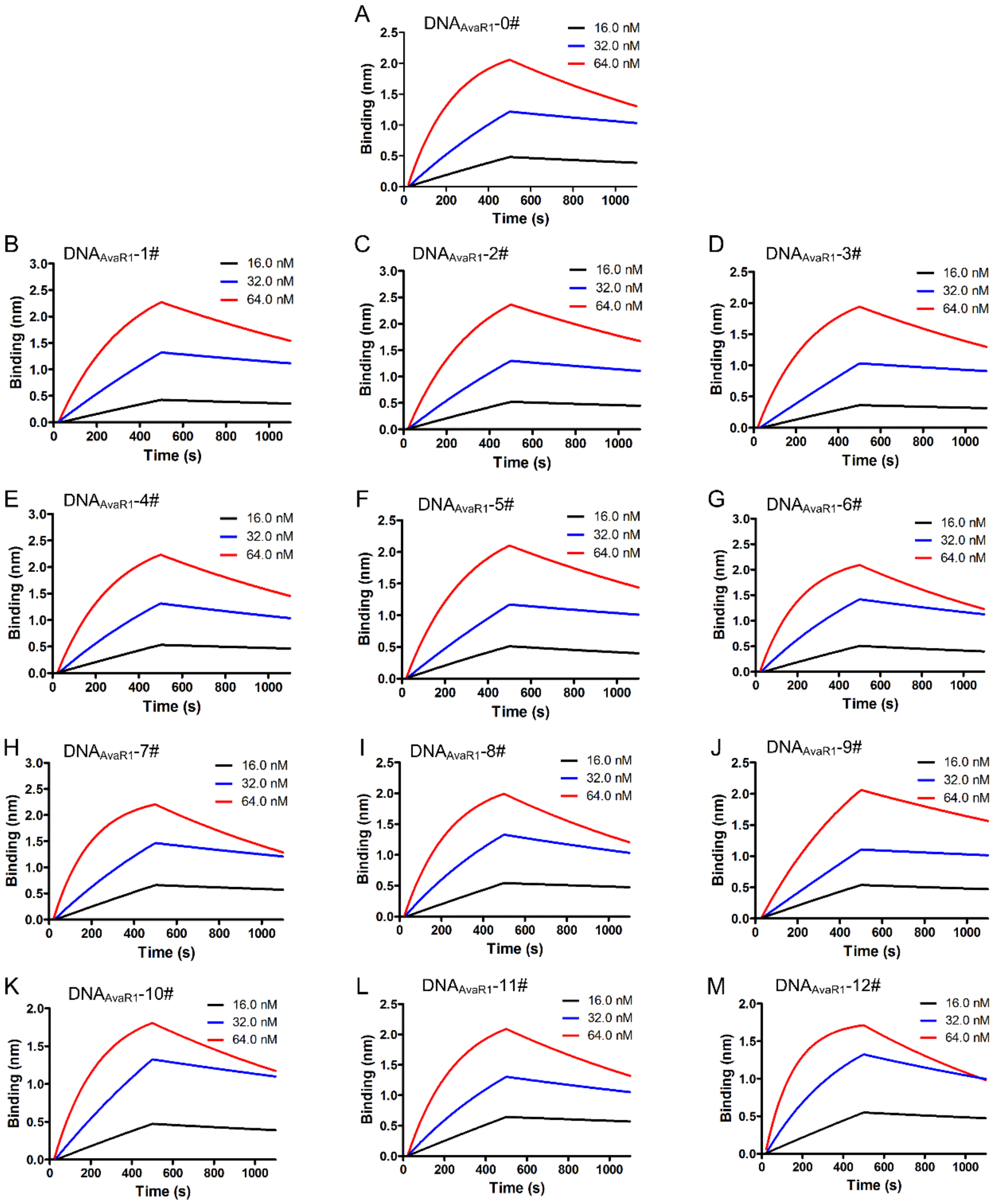
A
DNA_{AvaR1}-0#
B
DNA_{AvaR1}-1#
C
DNA_{AvaR1}-2#
D
DNA_{AvaR1}-3#
E
DNA_{AvaR1}-4#
F
DNA_{AvaR1}-5#
G
DNA_{AvaR1}-6#
H
DNA_{AvaR1}-7#
I
DNA_{AvaR1}-8#
J
DNA_{AvaR1}-9#
K
DNA_{AvaR1}-10#
L
DNA_{AvaR1}-11#
M
DNA_{AvaR1}-12#
16.0 nM
32.0 nM
64.0 nM
Binding (nm)
Time (s)

Fig. S5. Interaction kinetics of AvaR1 and its intact or nicked TFBSs. (**A**) Sensorgram of the interaction between AvaR1 and intact TFBS (DNA_{AvaR1}-0) determined by BLI. (**B**), (**C**), (**D**), (**E**), (**F**), (**G**), (**H**), (**I**), (**J**), (**K**), (**L**) and (**M**) show sensorgrams of the interaction between AvaR1 and its TFBSs with a nick at various positions (DNA_{AvaR1}-N, N indicates nicked position in Fig. 2C). 0.8 mM of DNA_{AvaR1}-N was used for loading step. The values of K_D, k_{on} and k_{off} are listed in table S4.

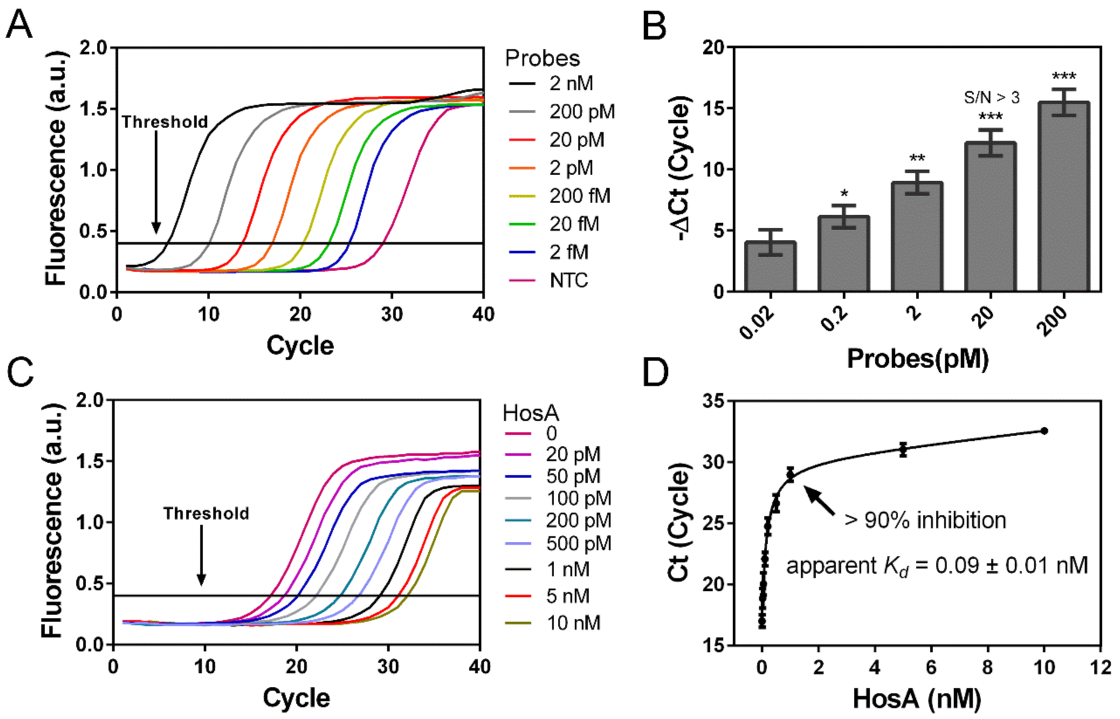

Fig. S6. Optimizing the system for combining aTF-NAST with RT-qPCR. (**A**) Amplification plot of different concentrations of probes for RT-qPCR assay. The concentration of T4 DNA ligase was 0.1 U/μL, and the probes contains probe-A, -B and -C with the ratio of 1:1:1. NTC was non-template control. (**B**) The relationship between Ct and -lg of probes (Probe-A, -B and -C) concentrations. Here 20 pM probes (Probe-A, -B and -C) could give satisfied ratio of signal to noise (S/N > 3). Significance analysis was conducted by one-way ANOVA. Significant difference was labelled with asterisk (*** $p < 0.001$, ** $p < 0.01$, * $p < 0.05$). (**C**) Amplification plot of samples with different amount of HosA. (**D**) The relationship between Ct and HosA concentrations. 1 nM of HosA was determined to use in the 4-HBA biosensor. Each reaction was run in triplicate and the curve shown is the average of the three repeats. For (**B**) and (**D**), results were expressed as mean ± s.d.

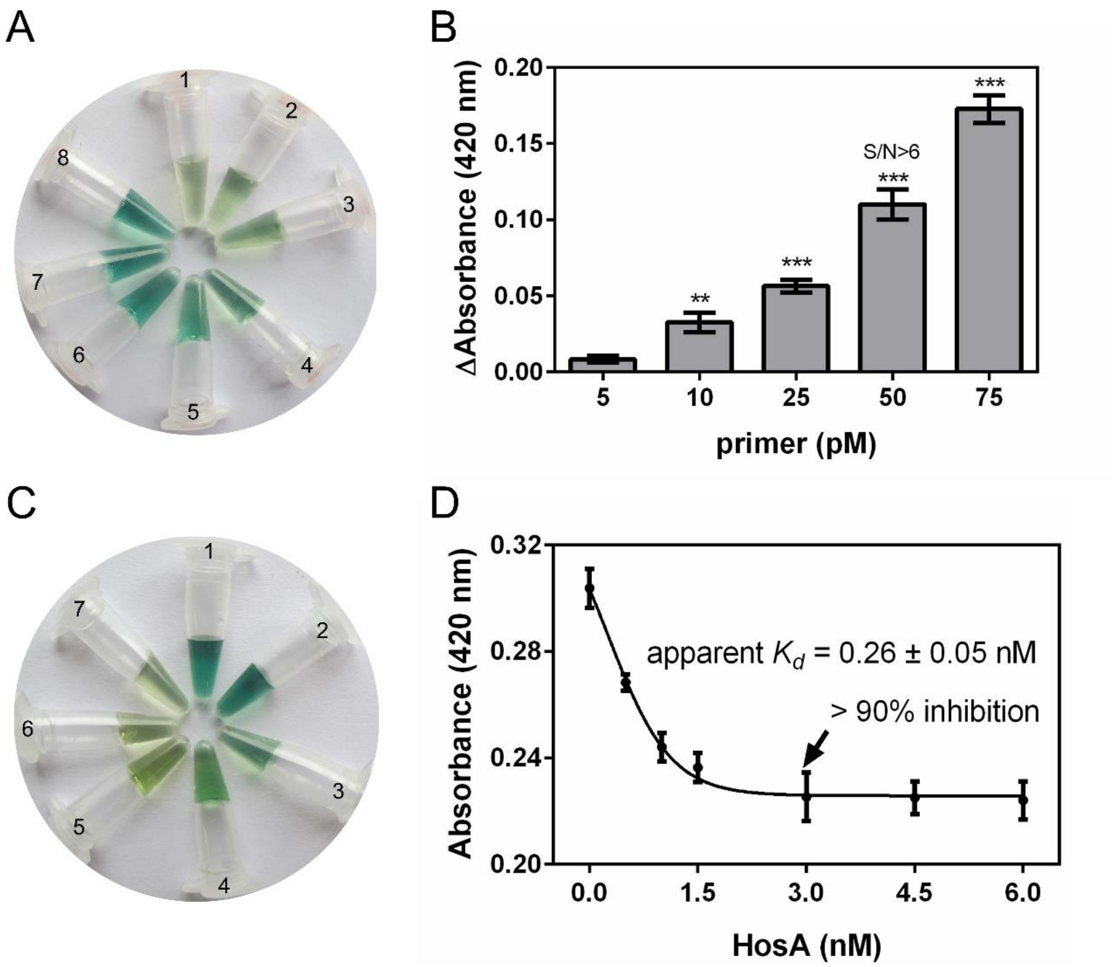
A
B
C
D
ΔAbsorbance (420 nm)
0.20
0.15
0.10
0.05
0.00
**
***
S/N>6
***
***
5
10
25
50
75
primer (pM)
Absorbance (420 nm)
0.32
0.28
0.24
0.20
apparent K_d = 0.26 ± 0.05 nM
> 90% inhibition
0.0
1.5
3.0
4.5
6.0
HosA (nM)

Fig. S7. Optimizing the system for combining aTF-NAST with RCA. (**A**) Optimizing the concentration of the primer using visible output determined by the activity of peroxidase-mimic G-quadruplexes. For each tube, the concentrations of template and T4 DNA ligase were 100 nM and 0.1 U/μL, respectively. Tubes 1, 2, 3, 4, 5, 6, 7 and 8 were 5, 10, 25, 50, 75, 100, 150 and 200 pM of the primers in the reactions, respectively. (**B**) Quantification of the activity of peroxidase-mimic G-quadruplexes by colorimetric analysis. 50 pM of primer could give satisfied S/N (S/N > 6). Significance analysis was conducted by one-way ANOVA. Significant difference was labelled with asterisk (*** $p < 0.001$, ** $p < 0.01$, * $p < 0.05$). (**C**) Optimizing the concentration of HosA for the inhibition of RCA output. The concentrations of HosA in tube 1, 2, 3, 4 and 5 were 0, 0.5, 1, 1.5, 3, 4.5 and 6 nM, respectively. (**D**) Quantification of the activity of peroxidase-mimic G-quadruplexes when adding different dosage of HosA. 3 nM of HosA could completely repress the RCA reaction. For (**B**) and (**D**), results were expressed as mean ± s.d.

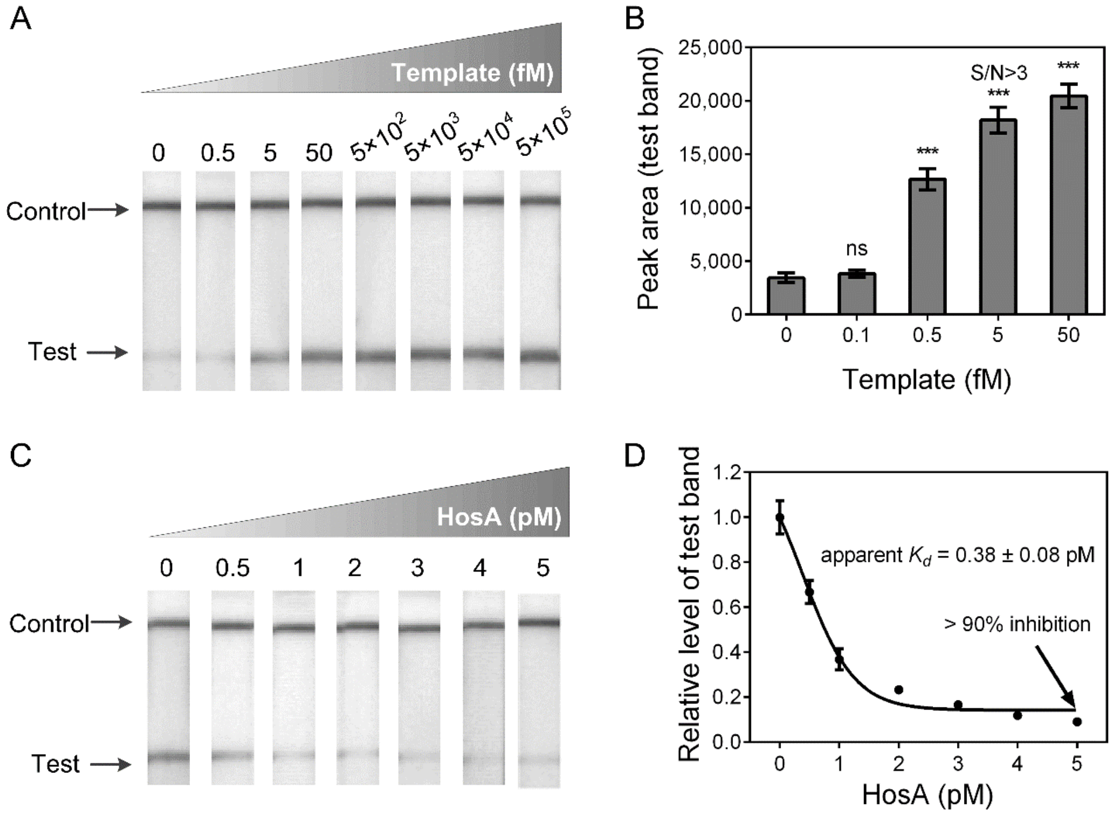

Fig. S8. Optimizing the system for combining aTF-NAST with RPA. (**A**) Determination of the optimal DNA template for LF assay. (**B**) Calibration curve of the relative level of outputs generated by different test bands. 5 fM of template was chosen for further studies because it provided a significant test band. Significance analysis was conducted by one-way ANOVA. Significant difference was labelled with asterisk (*** $p < 0.001$, ** $p < 0.01$, * $p < 0.05$). (**C**) Determination of the optimal concentration of HosA for the inhibition of RPA output. (**D**) Calibration curve of relative grey-scale intensity of test bands with HosA concentrations. 5 pM of HosA was adopted because it was the lowest concentration required for the inhibition of RPA reaction. For (**B**) and (**D**), data were the relative grey-scale intensity of the test bands determined by Image J software. The peak area of test band in the first strip was set to one.

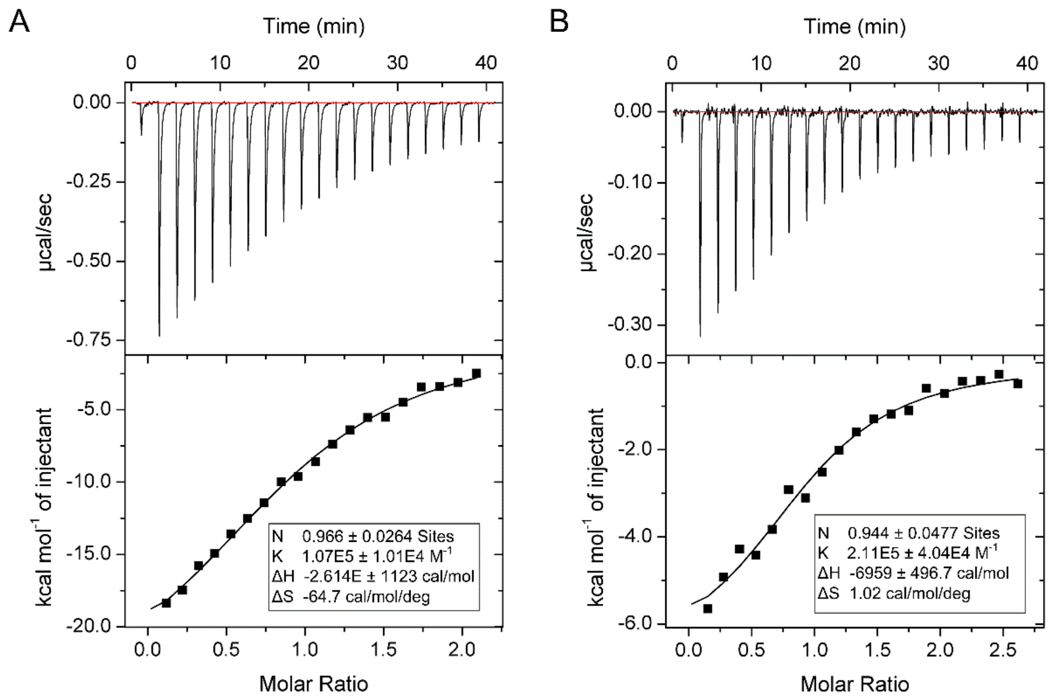

Fig. S9. Interaction between aTFs and corresponding small molecules determined by ITC. (**A**) Interaction between HucR and UA. UA (250 µM) was injected into 25 µM HucR diluted in ITC buffer. The K_D was determined to be 9.35 ± 0.99 µM. (**B**) Interaction between HosA and 4-HBA. 4-HBA (250 µM) was injected into 20 µM HosA diluted in ITC buffer. The K_D was determined to be 4.74 ± 0.25 µM. (Upper) Heat changes measured as a function of time at 25°C are shown. (Lower) Normalized heat changes (black square) and the best-fit curve (solid line) and calculated parameters are shown. (Inset) The estimated binding stoichiometry constant and enthalpy are shown.

Supplementary Tables

Table S1. Primers and oligonucleotides used in this work.

Primer		Sequence [a]
Usage	Name	
EMSA	E1	ATAACGTTCGTATACGAACCGCATTCAGGATGCGTAAGTCCTA
	E2	TAGGACTTACGCATCCTGAATGCGGTTCGTATACGAACGTTAT
Preparation of the intact DNA_{HosA}	DNA_{HosA} -F0	Biotin-CAGAGGCGTATTTTAATAATAACGTTCGTATACGAACAATTAAGAGAATAAACA TGAG
	DNA_{HosA}-R0	CTCATTGTTTATTCTCTTAATTGTTCGTATACGAACGTTATTATTAAAATACGCCTCTG
Preparation of a series of nicked DNA_{HosA}	DNA_{HosA}-R1-1	CTCATTGTTTATTCTCTTAATTGTTCGTATACGAACG
	DNA_{HosA}-R1-2	TTATTATTAAAATACGCCTCTG
	DNA_{HosA}-R2-1	CTCATTGTTTATTCTCTTAATTGTTCGTATACGA
	DNA_{HosA}-R2-2	ACGTTATTATTAAAATACGCCTCTG

	DNA_{HosA}-R3-1	CTCATTGTTTATTCTCTTAATTGTTCGTATAC
	DNA_{HosA}-R3-2	GAACGTTATTATTAAAATACGCCTCTG
	DNA_{HosA}-R4-1	CTCATTGTTTATTCTCTTAATTGTTCGTAT
	DNA_{HosA}-R4-2	ACGAACGTTATTATTAAAATACGCCTCTG
	DNA_{HosA}-R5-1	CTCATTGTTTATTCTCTTAATTGTTCGTA
	DNA_{HosA}-R5-2	TACGAACGTTATTATTAAAATACGCCTCTG
	DNA_{HosA}-R6-1	CTCATTGTTTATTCTCTTAATTGTTCGT
	DNA_{HosA}-R6-2	ATACGAACGTTATTATTAAAATACGCCTCTG
	DNA_{HosA}-R7-1	CTCATTGTTTATTCTCTTAATTGTTC
	DNA_{HosA}-R7-2	GTATACGAACGTTATTATTAAAATACGCCTCTG
	DNA_{HosA}-R8-1	CTCATTGTTTATTCTCTTAATTGT
	DNA_{HosA}-R8-2	TCGTATACGAACGTTATTATTAAAATACGCCTCTG
	DNA_{HosA}-R9-1	CTCATTGTTTATTCTCTTAA
	DNA_{HosA}-R9-2	TTGTTCGTATACGAACGTTATTATTAAAATACGCCTCTG
Preparation of the intact DNA_{TetR}	DNA_{TetR}-F0	Biotin-CCTCCGATTTTCAGATTGACACTCTATCATTGATAGGGATATATTCCAACTCTAT CAAT

	DNA_{TetR}-R0	ATTGATAGAGTTGGAATATATCCCTATCAATGATAGAGTGTCAATCTGAAAATCGGAGG
Preparation of a series of nicked DNA_{TetR}	DNA_{TetR}-R1-1	ATTGATAGAGTTGGAATATATCCCTATCAATGATAGAGTG
	DNA_{TetR}-R1-2	TCAATCTGAAAATCGGAGG
	DNA_{TetR}-R2-1	ATTGATAGAGTTGGAATATATCCCTATCAATGATAGAG
	DNA_{TetR}-R2-2	TGTCAATCTGAAAATCGGAGG
	DNA_{TetR}-R3-1	ATTGATAGAGTTGGAATATATCCCTATCAATGATAG
	DNA_{TetR}-R3-2	AGTGTCAATCTGAAAATCGGAGG
	DNA_{TetR}-R4-1	ATTGATAGAGTTGGAATATATCCCTATCAATGAT
	DNA_{TetR}-R4-2	AGAGTGTCAATCTGAAAATCGGAGG
	DNA_{TetR}-R5-1	ATTGATAGAGTTGGAATATATCCCTATCAATG
	DNA_{TetR}-R5-2	ATAGAGTGTCAATCTGAAAATCGGAGG
	DNA_{TetR}-R6-1	ATTGATAGAGTTGGAATATATCCCTATCAA
	DNA_{TetR}-R6-2	TGATAGAGTGTCAATCTGAAAATCGGAGG
	DNA_{TetR}-R7-1	ATTGATAGAGTTGGAATATATCCCTATCA
	DNA_{TetR}-R7-2	ATGATAGAGTGTCAATCTGAAAATCGGAGG

	DNA$_{\mathrm{TetR}}$-R8-1	ATTGATAGAGTTGGAATATATCCCTAT
	DNA$_{\mathrm{TetR}}$-R8-2	CAATGATAGAGTGTCAATCTGAAAATCGGAGG
	DNA$_{\mathrm{TetR}}$-R9-1	ATTGATAGAGTTGGAATATATCCCT
	DNA$_{\mathrm{TetR}}$-R9-2	ATCAATGATAGAGTGTCAATCTGAAAATCGGAGG
	DNA$_{\mathrm{TetR}}$-R10-1	ATTGATAGAGTTGGAATATATCC
	DNA$_{\mathrm{TetR}}$-R10-2	CTATCAATGATAGAGTGTCAATCTGAAAATCGGAGG
	DNA$_{\mathrm{TetR}}$-R11-1	ATTGATAGAGTTGGAATATAT
	DNA$_{\mathrm{TetR}}$-R11-2	CCCTATCAATGATAGAGTGTCAATCTGAAAATCGGAGG
	DNA$_{\mathrm{TetR}}$-R12-1	ATTGATAGAGTTGGAATAT
	DNA$_{\mathrm{TetR}}$-R12-2	ATCCCTATCAATGATAGAGTGTCAATCTGAAAATCGGAGG
Preparation of the intact DNA$_{\mathrm{AvaR1}}$	DNA$_{\mathrm{AvaR1}}$-F0	Biotin-CGCAAGAGGGACTTGAAGACAAAACCGTCTAGTACGTATCTTTGACCTCCAGCTCTTCC
	DNA$_{\mathrm{AvaR1}}$-R0	GGAAGAGCTGGAGGTCAAAGATACGTACTAGACGGTTTTGTCTTCAAGTCCCTCTTGCG
Preparation of a series of nicked	DNA$_{\mathrm{AvaR1}}$-R1-1	GGAAGAGCTGGAGGTCAAAGATACGTACTAGACGGTTTTG
	DNA$_{\mathrm{AvaR1}}$-R1-2	TCTTCAAGTCCCTCTTGCG

DNA_{AvaR1}	DNA_{AvaR1}-R2-1	GGAAGAGCTGGAGGTCAAAGATACGTACTAGACGGTTT
	DNA_{AvaR1}-R2-2	TGTCTTCAAGTCCCTCTTGCG
	DNA_{AvaR1}-R3-1	GGAAGAGCTGGAGGTCAAAGATACGTACTAGACGGT
	DNA_{AvaR1}-R3-2	TTTGTCTTCAAGTCCCTCTTGCG
	DNA_{AvaR1}-R4-1	GGAAGAGCTGGAGGTCAAAGATACGTACTAGACG
	DNA_{AvaR1}-R4-2	GTTTTGTCTTCAAGTCCCTCTTGCG
	DNA_{AvaR1}-R5-1	GGAAGAGCTGGAGGTCAAAGATACGTACTAGA
	DNA_{AvaR1}-R5-2	CGGTTTTGTCTTCAAGTCCCTCTTGCG
	DNA_{AvaR1}-R6-1	GGAAGAGCTGGAGGTCAAAGATACGTACTA
	DNA_{AvaR1}-R6-2	GACGGTTTTGTCTTCAAGTCCCTCTTGCG
	DNA_{AvaR1}-R7-1	GGAAGAGCTGGAGGTCAAAGATACGTAC
	DNA_{AvaR1}-R7-2	TAGACGGTTTTGTCTTCAAGTCCCTCTTGCG
	DNA_{AvaR1}-R8-1	GGAAGAGCTGGAGGTCAAAGATACGT
	DNA_{AvaR1}-R8-2	ACTAGACGGTTTTGTCTTCAAGTCCCTCTTGCG
	DNA_{AvaR1}-R9-1	GGAAGAGCTGGAGGTCAAAGATAC
	DNA_{AvaR1}-R9-2	GTACTAGACGGTTTTGTCTTCAAGTCCCTCTTGCG
	DNA_{AvaR1}-R10-1	GGAAGAGCTGGAGGTCAAAGAT

	DNA_{AvaR1}-R10-2	ACGTACTAGACGGTTTTGTCTTCAAGTCCCTCTTGCG
	DNA_{AvaR1}-R11-1	GGAAGAGCTGGAGGTCAAAG
	DNA_{AvaR1}-R11-2	ATACGTACTAGACGGTTTTGTCTTCAAGTCCCTCTTGCG
	DNA_{AvaR1}-R12-1	GGAAGAGCTGGAGGTCAA
	DNA_{AvaR1}-R12-2	AGATACGTACTAGACGGTTTTGTCTTCAAGTCCCTCTTGCG
d-PAGE	T_H	CAATTAAGCTTAATTGTTCGTATACGAACGTTATTA
	T_L	GACTACACCAAGGGCTACAAGGTGTATCTTCATACGAACGTTTAATAACGTTCGTATACG
	T_R	pAACAATTAAGCTTAATTGTTCGTACTCACATGGTCGAGGTCATCAA
	T_{L+R}	GACTACACCAAGGGCTACAAGGTGTATCTTCATACGAACGTTTAATAACGTTCGTATACGAACAATTAAGCTTAATTGTTCGTACTCACATGGTCGAGGTCATCAA
Biosensors with RT-qPCR output	HBA-Probe-A	GACTACACCAAGGGCTACAAGGTGTATCTTCATACGAACGTTTAATAACGTTCGTATACG
	HBA-Probe-B	p-AACAATTAAGCTTAATTGTTCGTACTCACATGGTCGAGGTCATCAA
	HBA-Probe-C	TAATAACGTTCGTATACGAACAATTAAGCTTAATTG-p

	UA-Probe-A	GACTACACCAAGGGCTACAAGGTGTATCTTCATACGAACGTTTAATAATACTTAGATGTCTA
	UA-Probe-B	p- CCTAAATTAAGCTTAATTGTTCGTACTCACATGGTCGAGGTCATCAA
	UA-Probe-C	CAATTAAGCTTAATTTAGGTAGACATCTAAGTATTATTA-p
	TC-Probe-A	GACTACACCAAGGGCTACAAGGTGTATCTTCATACGAACGTTTAATAAACTCTATCATTGATA
	TC-Probe-B	p-GAGTAATTAAGCTTAATTGTTCGTACTCACATGGTCGAGGTCATCAA
	TC-Probe-C	CAATTAAGCTTAATTACTCTATCAATGATAGAGTTTATTA-p
	Forward	TTGATGACCTCGACCATGTG
	Reverse	GACTACACCAAGGGCTACAAG
Biosensors with RCA output	HBA-CT	p-AACAAAAA*CCTCAGC* **CCAACCCGCCCTACCCAC** *CCTCAGC* **CCAACCCGCCCTACCCAC** *CCTCAGC*CGTTCGTATACG
	HBA-P	TGAGGTTTTTGTTCGTATACGAACG
	UA-CT	p-GTAAAAAA*CCTCAGC* **CCAACCCGCCCTACCCAC** *CCTCAGC* **CCAACCCGCCCTACCCAC** *CCTCAGC*CTAGGTAGACATCTAA
	UA-P	TGAGGTTTTTTACTTAGATGTCTACCTA

	TC-CP	p-AGGAAAAA*CCTCAGC***CCAACCCGCCCTACCCAC***CCTCAGC***CCAACCCGCCCTACC** **CAC***CCTCAGC*CTCTATCATTGAT
	TC-P	TGAGGTTTT TCCTATCAATGATAGA
	HRP	GTGGGTAGGGCGGGTTGG
Biosensors with RPA output	HBA-A	p-GCATATGCTTGCAATAATTTGCAAGCATACTTCTATGTGGAACATCGGGAACCACAT CAGTATCATCGCCGTCTATCAAACGGGCCTCAAATGTACCGCAATGCTTTATTTC
	HBA-B	CGGGGACAGTAGGGCTGCTAGTCAGAGTAACAAATAAGAGAATTAACTACTGGAGCTG GTACACTCATGCTTGTTAATTCGAATTAACAA
	HBA-C	CTTGCAAATTATTGCAAGCATATGCTTGTTAATTCGAATTAACAAGCATG-p
	UA-A	p-AATCTACAGATGGATCAATAATTTGCAAGCATACTTCTATGTGGAACATCGGGAACC ACATCAGTATCATCGCCGTCTATCAAACGGGCCTCAAATGTACCGCAATGCTTTATTTC
	UA-B	CGGGGACAGTAGGGCTGCTAGTCAGAGTAACAAATAAGAGAATTAACTACTGGAGCTG GTACACTCATGCTTGTTAATTCGAATTAAATG
	UA-C	CTTGCAAATTATTGATCCATCTGTAGATTCATTTAATTCGAATTAACAAGCATG-p
	TC-A	p-ATCATTGATAGGCAATAATTTGCAAGCATACTTCTATGTGGAACATCGGGAACCACA TCAGTATCATCGCCGTCTATCAAACGGGCCTCAAATGTACCGCAATGCTTTATTTC

TC-B	CGGGGACAGTAGGGCTGCTAGTCAGAGTAACAAATAAGAGAATTAACTACTGGAGCTG GTACACTCATGCTTGTTAATTCGAATTAATCT
TC-C	CTTGCAAATTATTG CCTATCAATGATAGA TTAATTCGAATTAACAAGCATG-p
RPA-P1	GAAATAAAGCATTGCGGTACATTTGAGGCCCGTTT
RPA-P2	Biotin-CGGGGACAGTAGGGCTGCTAGTCAGAGTAACAAAT
RPA-Probe	FAM-GATAGACGGCGATGATACTGATGTGGTTCCCGATG[dSpacer]TTCCACATAGAAGT ATG-ddC

[a] The fragment in red is the TFBS. The black bold sequence is complementary with the fragment which can fold into DNAzyme. And the italic letters is the restriction site of Nb.BbvCI. dd indicates dideoxy. The –p at 3’ end indicates a phosphorylated modification.

Table S2. Parameters of the interaction dynamics between HosA and intact or nicked TFBSs.

Probe	K_D (nM) [a]	k_{on} (1/Ms) ×10^6	k_{off} (1/s) ×10^{-4}	R^2 value
DNA_{HosA}-0#	13.01 ± 0.02	1.27 ± 0.02	16.51 ± 0.11	0.9548
DNA_{HosA}-1#	6.84 ± 0.01	1.34 ± 0.02	8.52 ± 0.06	0.9607
DNA_{HosA}-2#	7.92 ± 0.01	1.28 ± 0.02	9.45 ± 0.07	0.958
DNA_{HosA}-3#	9.22 ± 0.01	1.11 ± 0.01	9.53 ± 0.07	0.9614
DNA_{HosA}-4#	10.34 ± 0.02	1.14 ± 0.02	10.98 ± 0.08	0.9635
DNA_{HosA}-5#	12.34 ± 0.02	0.93 ± 0.01	10.67 ± 0.08	0.9625
DNA_{HosA}-6#	9.32 ± 0.01	1.12 ± 0.02	9.67 ± 0.09	0.9633
DNA_{HosA}-7#	8.15 ± 0.02	1.32 ± 0.01	10.07 ± 0.07	0.9603
DNA_{HosA}-8#	7.46 ± 0.01	1.28 ± 0.02	9.55 ± 0.08	0.9649
DNA_{HosA}-9#	9.33 ± 0.02	1.11 ± 0.01	10.35 ± 0.09	0.9583

The constants "k_{on}", "k_{off}", and "K_D" are fitted by built-in equations of BLI machine (Fortébio). [a] Affinity of HosA and different DNA_{TFBS} was measured by BLI and K_D was calculated using a 1:1 model ($R^2 > 0.95$).

Table S3. Parameters of the interaction dynamics between TetR and intact or nicked TFBSs.

Probe	K_D (nM) [a]	k_{on} (1/Ms) × 10^5	k_{off} (1/s) × 10^{-4}	R^2 value
DNA_{TetR} -0#	1.08 ± 0.06	3.33 ± 0.14	3.59 ± 0.18	0.9533
DNA_{TetR}-1#	1.30 ± 0.04	4.20 ± 0.07	5.46 ± 0.11	0.9681
DNA_{TetR}-2#	1.78 ± 0.07	3.20 ± 0.09	5.69 ± 0.14	0.9355*
DNA_{TetR}-3#	1.72 ± 0.11	3.22 ± 0.08	5.54 ± 0.14	0.9587
DNA_{TetR}-4#	1.89 ± 0.07	3.53 ± 0.11	6.67 ± 0.15	0.9635
DNA_{TetR}-5#	1.91 ± 0.03	1.95 ± 0.09	3.72 ± 0.19	0.9064*
DNA_{TetR}-6#	2.00 ± 0.59	1.86 ± 0.17	3.72 ± 0.19	0.9509
DNA_{TetR}-7#	1.85 ± 0.06	3.23 ± 0.07	5.97 ± 0.13	0.9579
DNA_{TetR}-8#	1.52 ± 0.044	4.37 ± 0.08	6.64 ± 0.11	0.9270*
DNA_{TetR}-9#	0.67 ± 0.03	3.26 ± 0.09	2.18 ± 0.14	0.9324*

DNA_{TetR}-10#	1.22 ± 0.03	4.07 ± 0.07	4.96 ± 0.11	0.9785
DNA_{TetR}-11#	1.58 ± 0.04	2.15 ± 0.08	3.39 ± 0.09	0.9729
DNA_{TetR}-12#	1.10 ± 0.05	1.89 ± 0.14	2.08 ± 0.15	0.9547

The constants “k_{on}”, “k_{off}”, and “K_D” are fitted by built-in equations of BLI machine (Fortébio). [a] Affinity of TetR and different DNA_{TFBS} was measured by BLI and K_D was calculated using a 1:1 model ($R^2 > 0.95$). Asterisk indicates $R^2 > 0.90$.

Table S4. Parameters of the interaction dynamics between AvaR1 and intact or nicked TFBSs.

Probe	K_D (nM) [a]	k_{on} (1/Ms)×10^4	k_{off} (1/s) ×10^{-4}	R^2 value
DNA_{AvaR1}-0#	11.70 ± 0.028	4.88 ± 0.096	5.68 ± 0.079	0.9807
DNA_{AvaR1}-1#	18.30 ± 0.052	2.80 ± 0.069	5.12 ± 0.063	0.9887
DNA_{AvaR1}-2#	15.10 ± 0.034	3.10 ± 0.059	4.68 ± 0.054	0.9913
DNA_{AvaR1}-3#	17.0 ± 0.052	2.55 ± 0.076	4.34 ± 0.038	0.9968
DNA_{AvaR1}-4#	16.00 ± 0.032	3.64 ± 0.064	5.81 ± 0.063	0.9910
DNA_{AvaR1}-5#	14.00 ± 0.027	3.53 ± 0.071	4.95 ± 0.076	0.9879
DNA_{AvaR1}-6#	12.60 ± 0.021	5.21 ± 0.094	6.54 ± 0.092	0.9837
DNA_{AvaR1}-7#	10.80 ± 0.030	7.54 ± 0.111	8.14 ± 0.069	0.9646
DNA_{AvaR1}-8#	14.10 ± 0.025	4.49 ± 0.083	6.31 ± 0.046	0.9860
DNA_{AvaR1}-9#	22.20 ± 0.245	3.86 ± 0.048	3.61 ± 0.046	0.9937

DNA $_{AvaR1}$-10#	11.00 ± 0.026	4.58 ± 0.087	5.04 ± 0.069	0.9856
DNA $_{AvaR1}$-11#	13.00 ± 0.027	4.42 ± 0.076	5.75 ± 0.063	0.9874
DNA $_{AvaR1}$-12#	8.33 ± 0.015	7.99 ± 0.114	6.65 ± 0.076	0.9815

The constants "k_{on}", "k_{off}", and "K_D" are fitted by built-in equations of BLI machine (Fortébio). [a] Affinity between AvaR1 and different DNA_{TFBS} was measured by BLI and K_D was calculated using a 1:1 model (R^2 > 0.95).

Table S5. Performance of the developed aTF-based biosensors in this study.

Analytes	aTFs	Combined detection method	LOD (nM)	Detection range (nM)
4-HBA	HosA	RT-qPCR	1.12	5 – 300
		RCA	1.73	5 – 200
		RPA	0.0005	0.001 – 10
UA	HucR	RT-qPCR	3.08	10 – 600
		RCA	3.96	10 – 200
		RPA	0.0002	0.0005 – 10
TC	TetR	RT-qPCR	1.73	5 – 600
		RCA	1.21	10 – 300
		RPA	0.005	0.01 – 1000

Table S6. Comparison with previously reported UA biosensors.

Recognition elements	Transduction system	Linear range (μM)	LOD (μM)	Time and references
Uricase	PPy-Fc/Pt	1–50	0.5	2006 (*43*)
Uricase	PANI-Ppy/Au	2.5–85	1	2008 (*44*)
Uricase	CuO/Pt/glass	50–1000	140	2012 (*45*)
Uricase	ZnO:N	50–1000	40	2013 (*46*)
Uricase	Graphite/$Ru(bpy)_3^{2+}$	10–1000	3.1	2013 (*47*)
Uricase	PTH/CNT/CFE	2–100	0.08	2014 (*48*)
Uricase	Ni/NiO/ITO/glass	50–1000	30	2014 (*49*)
Uricase	Chi-CNTsNF/AgNPs/Au	1–400	1	2014 (*50*)
Uricase	NiO/Pt/Ti/glass	50–1000	40	2014 (*51*)
Uricase	PdAg NFs/rGO/GCE	1–150	0.081	2015 (*52*)
Uricase	Naf/Fc/GCE	0.5–600	0.23	2015 (*53*)
Uricase	HRP-CdS quantum dots	125–1000	12	2015 (*54*)
Uricase	c-MWCNT/GEL/PVF/GCE	0.2–710	0.023	2015 (*55*)
Uricase	GSH-capped CdTe NPs	0.22–6	0.1	2016 (*56*)
Uricase	CS/HRP–PD/Au_{plate}/Au	1–1250	0.1	2016 (*57*)
Uricase	UCNPs/4-AAP/TOPS/$ZnFe_2O_4$	10–1000	2.86	2016 (*58*)
Uricase	S, N co-doped C-dots	0.08–10	0.07	2016 (*59*)
Uricase	BSA–AuNCs	10–800	6.6	2016 (*60*)
Uricase	Nafon/Uricase-ZNRs/Ag/glass	10–4560	0.005	2017 (*61*)
Uricase	TOPS/4-AAP	2.0–40	0.5	2017 (*62*)
Uricase	DNAzyme/GODs	2–300	0.5	2017 (*63*)

Uricase	CeO2-x/C/rGO Nanocomposites	49.8–1050.0	2	2018 (*64*)
Uricase	Mn^{2+}-doped $NaYF_4$:Yb,Er UCNPs	0.004–10	0.001	2018 (*65*)
Uricase	4-AAP/TOPS	10–1000	2	2018 (*66*)
HucR	aTF-NAST (RT-qPCR)	0.01–0.6	0.00308	This work
HucR	aTF-NAST (RCA)	0.01–0.2	0.00396	This work
HucR	aTF-NAST (RPA)	0.0000005–0.01	0.0000002	This work

Table S7. Comparison with previously reported TC biosensors.

Recognition elements	Transduction system	Linear range (nM)	LOD (nM) [a]	Time and references
TetR	Cell-free biosensor	14–23000	ND	2004 (*67*)
TetR	Whole-cell biosensor	11–36000	11	2005 (*68*)
Antibody	ELISA	3.4–342	0.43	2008 (*69*)
HPLC	UV	1.13–2250	ND	2009 (*70*)
Aptamer	Electrochemical	10 –10000	5	2012 (*71*)
Aptamer	Enzyme-linked aptamer	0.023–225	0.022	2014 (*72*)
Aptamer	HMS) and AuNPs	0.3–10	0.266	2015 (*73*)
Aptamer	Interdigital array microelectrode	1–1000	ND	2017 (*74*)
Aptamer	BiPO4/3DNGH PEC	0.1–1000	0.033	2018 (*75*)
TetR	aTF-NAST (RT-qPCR)	5–600	1.73	This work
TetR	aTF-NAST (RCA)	10–300	1.21	This work
TetR	aTF-NAST (RPA)	0.01–1000	0.005	This work

[a] ND indicates that the data is not provided in the corresponding report.

Table S8. Performance of the developed biosensors.

Analyte	Approach	Concentration[a]				Accuracy[b]				Precision[c]	Recovery[d]
4-HBA		0	1	5	10	0	1	5	10		
(nM)	HPLC	ND	0.98 ± 0.02	4.86 ± 0.09	9.83 ± 0.13	/	100	100	100	/	/
	RCA	ND	1.00 ± 0.10	5.06 ± 0.21	9.09 ± 0.44	/	101.7	104.2	92.5	6.16	105.61
	RPA	ND	1.07 ± 0.11	5.64 ± 0.47	10.17 ± 0.52	/	108.7	116.1	103.5	5.78	118.43
	RT-qPCR	ND	0.99 ± 0.04	4.70 ± 0.19	9.11 ± 0.13	/	100.8	96.8	92.7	4.18	98.47
UA		0	1	10	20	0	1	10	20		
(μM)	HPLC	203.21 ± 1.21	204.70 ±1.34	212.62 ±1.82	224.71 ± 1.75	100	100	100	100	/	/
	RCA	197.92 ± 2.02	209.61± 3.17	225.80 ± 3.53	208.31 ± 4.01	97.4	102.4	106.2	92.7	6.93	103.62
	RPA	190.00 ± 6.53	197.94 ± 5.22	186.04 ± 5.82	195.05 ± 4.61	93.5	96.7	87.5	86.8	6.11	83.68
	RT-qPCR	209.51 ± 2.34	207.77 ± 3.28	217.72 ± 2.97	241.79 ± 3.05	103.1	101.5	102.4	107.6	3.17	101.7
TC		0	1	2	5	0	1	2	5		
(nM)	HPLC	ND	0.91± 0.03	1.92 ± 0.02	4.87 ± 0.18	/	100	100	100	/	/
	RCA	ND	0.95 ± 0.10	2.09 ± 0.16	4.54 ± 0.14	/	104.1	108.7	93.3	7.75	105.72
	RPA	ND	0.88 ± 0.02	1.75 ± 0.02	4.36 ± 0.22	/	96.7	91.27	89.6	4.01	88.31

RT-qPCR	ND	0.89 ± 0.07	2.02 ± 0.05	4.61 ± 0.11	/	98.3	105.2	94.7	5.37	102.54

[a] Concentration was the data detected from real samples by our biosensors. Data were the vales from three replicates. Values without standard derivation were the spiked concentration to real sample. Negative controls were those with no spiked target small molecules. In the control, except UA in human serum (Beijing Solarbio Science & Technology Co., Ltd.), 4-HBA in tested river water (from Beijing Olympic park) and TC in Satine organic milk (Inner Mongolia Yili Industrial Group Co., Ltd) were not detectable. “ND” means not detectable.

[b] Accuracy (%) was calculated by normalizing the concentration determined by biosensors to that determined by HPLC. Accuracy% = $\frac{\text{Concentration determined by biosensor}}{\text{Concentration determined by HPLC}}$.

[c] Precision was assessed by the relative standard derivation (RSD%). RSD% = $\frac{\text{Standard derivation of accuracy}}{\text{Mean value of accuracy}} \times 100\%$.

[d] Recovery tests were implemented by adding extra target small molecules to the spiked samples with same concentrations, *eg*. adding extra 5 nM of 4-HBA to the original 5 nM 4-HBA spiked river water, so do the tests for different approaches and samples. Recovery% = $\frac{\text{Concentration of the extra spiked sample - Concentration of the original spiked sample}}{\text{Concentration of the original spiked sample}} \times 100\%$.

Table S9. Comparison of the performance of the developed UA biosensors in a clinical test.

Number of human serum sample	UA concentration (μM) [a]				
	HPLC [b]	Clinic Test [c]	aTF-NAST RT-qPCR	aTF-NAST RCA	aTF-NAST RPA
1	342	378	351	367	373
2	274	267	291	311	265
3	543	512	557	574	581
4	314	349	337	336	325
5	312	332	346	357	342
6	259	281	275	279	238
7	621	601	594	587	603
8	327	358	336	345	309
9	290	304	303	314	317
10	308	296	312	276	274
Precision (RSD)	/	6.49	3.94	7.32	7.78

[a] UA concentration was the average of three measurements.

[b] HPLC results were used as standard results.

[c] Automatic biochemistry analyzer (Roche) was used in clinic test.
